# A Survey of Nucleotide Cyclases in Actinobacteria: Unique Domain Organization and Expansion of the Class III Cyclase Family in *Mycobacterium tuberculosis*

**DOI:** 10.1002/cfg.349

**Published:** 2004-02

**Authors:** Avinash R. Shenoy, K. Sivakumar, A. Krupa, N. Srinivasan, Sandhya S. Visweswariah

**Affiliations:** 1 Department of Molecular Reproduction Development and Genetics Indian Institute of Science Bangalore 560012 India; 2 Molecular Biophysics Unit Indian Institute of Science Bangalore 560012 India

## Abstract

Cyclic nucleotides are well-known second messengers involved in the regulation of important metabolic pathways or virulence factors. There are six different classes of nucleotide cyclases that can accomplish the task of generating cAMP, and four of these are restricted to the prokaryotes. The role of cAMP has been implicated in the virulence and regulation of secondary metabolites in the phylum Actinobacteria, which contains important pathogens, such as *Mycobacterium tuberculosis, M. leprae, M. bovis* and *Corynebacterium*, and industrial organisms from the genus *Streptomyces*. We have analysed the actinobacterial genome sequences found in current databases for the presence of different classes of nucleotide cyclases, and find that only class III cyclases are present in these organisms. Importantly, prominent members such as *M. tuberculosis* and *M. leprae* have 17 and 4 class III cyclases, respectively, encoded in their genomes, some of which display interesting domain fusions seen for the first time. In addition, a pseudogene corresponding to a cyclase from *M. avium* has been identified as the only cyclase pseudogene in *M. tuberculosis* and *M. bovis*. The *Corynebacterium* and *Streptomyces* genomes encode only a single adenylyl cyclase each, both of which have corresponding orthologues in *M. tuberculosis*. A clustering of the cyclase domains in Actinobacteria reveals the presence of typical eukaryote-like, fungi-like and other bacteria-like class III cyclase sequences within this phylum, suggesting that these proteins may have significant roles to play in this important group of organisms.


**Comparative and Functional Genomics**
*Comp Funct Genom* 2004; **5**: 17–38.
Published online in Wiley InterScience (www.interscience.wiley.com). **DOI:** 10.1002/cfg.349

**Primary Research Paper**

### A survey of nucleotide cyclases in Actinobacteria: unique domain organization and expansion of the class III cyclase family in *Mycobacterium tuberculosis*

Avinash R. Shenoy[1]**, K. Sivakumar[2], A. Krupa[2], N. Srinivasan[2] and Sandhya S. Visweswariah[1]*

[1] *Department of Molecular Reproduction, Development and Genetics, Indian Institute of Science, Bangalore 560012, India*
[2] *Molecular Biophysics Unit, Indian Institute of Science, Bangalore 560012, India*

**Correspondence to: Sandhya S. Visweswariah, Department of Molecular Reproduction, Development and Genetics, Indian Institute of Science, Bangalore 560012, India. E-mail: sandhya@mrdg.iisc.ernet.in*

***Correspondence to: Avinash R. Shenoy, Department of Molecular Reproduction, Development and Genetics, Indian Institute of Science, Bangalore 560012, India. E-mail: avirs@mrdg.lisc.ernet.in*

Received: 7 August 2003
Revised: 13 October 2003
Accepted: 21 October 2003

### Abstract

**Cyclic nucleotides are well-known second messengers involved in the regulation of important metabolic pathways or virulence factors. There are six different classes of nucleotide cyclases that can accomplish the task of generating cAMP, and four of these are restricted to the prokaryotes. The role of cAMP has been implicated in the virulence and regulation of secondary metabolites in the phylum Actinobacteria, which contains important pathogens, such as *Mycobacterium tuberculosis, M. leprae, M. bovis* and *Corynebacterium,* and industrial organisms from the genus *Streptomyces*. We have analysed the actinobacterial genome sequences found in current databases for the presence of different classes of nucleotide cyclases, and find that only class III cyclases are present in these organisms. Importantly, prominent members such as *M. tuberculosis* and *M. leprae* have 17 and 4 class III cyclases, respectively, encoded in their genomes, some of which display interesting domain fusions seen for the first time. In addition, a pseudogene corresponding to a cyclase from *M. avium* has been identified as the only cyclase pseudogene in *M. tuberculosis* and *M. bovis.* The *Corynebacterium* and *Streptomyces* genomes encode only a single adenylyl cyclase each, both of which have corresponding orthologues in *M. tuberculosis.* A clustering of the cyclase domains in Actinobacteria reveals the presence of typical eukaryote-like, fungi-like and other bacteria-like class III cyclase sequences within this phylum, suggesting that these proteins may have significant roles to play in this important group of organisms. Copyright © 2004 John Wiley & Sons, Ltd.**

**Keywords: class III cyclase; adenylyl cyclase; genome analysis; comparative genomics; Actinobacteria; *Mycobacterium tuberculosis*; *Mycobacterium leprae***

### Introduction

Cyclic AMP and cGMP are involved in diverse signalling networks in all life forms. In bacteria, cAMP is an important second messenger that regulates several operons and regulons (Cases *et al.* 1998). There are six different families of proteins that convert NTPs to either cAMP or cGMP (Danchin 1993; Shenoy *et al.* 2002), and these are distinguished by differences in amino acid sequence and catalytic mechanism, suggesting that these six classes have evolved independently in diverse organisms during evolution.

The class I enzymes are present in *E. coli* and related enteric bacteria (Danchin 1993), and are represented by single copy genes that code for an adenylyl cyclase involved in the phenomenon of catabolite repression (Cases *et al.* 1998). Class II cyclases are represented by toxins secreted by *Bacillus anthracis* (Leppla 1982), *Bordetella pertussis* (Weiss *et al.*, 1984) and *Pseudomonas*

Copyright © 2004 John Wiley & Sons, Ltd.

*aeruginosa* (Yahr *et al.*, 1998) that elevate cAMP levels within the host cells on infection. The crystal structure of the anthrax oedema factor, the calmodulin-activated adenylyl cyclase, shows that the catalytic mechanism of the class II cyclases appears to require a single divalent metal ion (Drum *et al.*, 2002).

The class III enzymes are the most widely distributed and are found in bacteria, archaea and eukaryotes (Danchin, 1993; Shenoy *et al.*, 2002). The mammalian G-protein coupled, receptor-activated adenylyl cyclases are members of the class III adenylyl cyclase family (Defer *et al.*, 2000), as are the receptor and soluble guanylyl cyclases (Wedel *et al.*, 2001). Crystal structures of the class III adenylyl cyclases (Tesmer *et al.*, 1997; Bieger *et al.*, 2001) and subsequent homology modelling and mutational analysis of the guanylyl cyclases (Liu *et al.*, 1997) have led to the identification of amino acid residues responsible for substrate selectivity amongst these enzymes (Sunahara *et al.*, 1998; Tucker *et al.*, 1998; Hannenhalli *et al.*, 2000). Classes IV, V and VI of the cyclase family have only a single representative each, found in *Aeromonas* (Sismeiro *et al.*, 1998), *Prevotella* (Cotta *et al.*, 1998) and *Rhizobium* (Tellez-Sosa *et al.*, 2002), respectively.

Class III cyclases have been the most extensively studied, both structurally and biochemically. The mammalian 12-transmembrane adenylyl cyclases act as functional dimers of the C1 and C2 regions contained within a single polypeptide chain (Tang *et al.*, 1998). The C1 and C2 regions are class III cyclase domains, hence mammalian membrane-bound adenylyl cyclases have two class III domains within their polypeptide chains. Crystal structures have revealed that the C1–C2 dimer interface gives rise to the active site, and hence dimerization and correct juxtaposition of residues from both protomers is essential for activity of these enzymes (Tang *et al.*, 1998). The guanylyl cyclases, on the other hand, have only a single class III cyclase domain per polypeptide and act by homodimerization, as is seen in the receptor guanylyl cyclases, or as heterodimers of $\alpha$ and $\beta$ subunits in the case of the soluble guanylyl cyclases (Wedel *et al.*, 2001). Mutagenesis studies (Zimmermann *et al.*, 1998) and structural similarity of the adenylyl cyclase core to the palm fold of DNA polymerases suggested that the cyclase reaction mechanism involves two-metal ion catalysis (Tesmer *et al.*, 1998). Two metal atoms are bound by two conserved aspartate residues, and the reaction involves the abstraction of the proton from the 3′ OH group of ATP and the nucleophilic attack by the negatively charged oxygen on the phosphodiester bond (Tesmer *et al.*, 1997). The abstraction of the proton is facilitated by one of the two metal atoms in the class III cyclases (Tesmer *et al.*, 1997), and this function is interestingly carried out by a suitably placed histidine residue in the class II cyclases (Drum *et al.*, 2002). A pentavalent phosphate group is believed to be the transition state species that is stabilized by critical asparagine and arginine residues, which have been shown to be essential for activity by mutational analysis (Tesmer *et al.*, 1997; Yan *et al.*, 1997). The binding of ATP requires a lysine and an aspartate, which are replaced by glutamate and cysteine in the guanylyl cyclases to allow utilization of GTP as substrate (Liu *et al.*, 1997).

The Actinobacteria include several bacteria of medical or industrial interest. *Streptomyces* is a ubiquitous soil organism known for its versatile metabolic prowess, especially in forming useful secondary metabolites, the production of some being regulated by cAMP (Susstrunk *et al.*, 1998; Horinouchi *et al.*, 2001). *Mycobacterium tuberculosis* is the aetiological agent of tuberculosis and belongs to an order that includes other pathogens such as *M. leprae* and several members in other genera, such as *Corynebacterium, Nocardia* and *Actinomyces* (NCBI **http://www.ncbi.nlm.nih.gov/entrez/query.fcgi?db=Taxonomy**). Interestingly, *M. tuberculosis* infection of macrophages has been shown to lead to increased cAMP in macrophages (Lowrie *et al.*, 1979) and *M. microti* has been thought to escape macrophage killing by the release of cAMP (Lowrie *et al.*, 1975). Since studies with *Bordetella pertussis* (Confer *et al.*, 1982), *Bacillus anthracis* (Hoover *et al.*, 1994) and *Trypanosoma* (Wirth *et al.*, 1982) have demonstrated the importance of cAMP in reducing phagocyte activity, it would be interesting to investigate whether cAMP plays any role in compromising the functioning of the macrophage in the case of *Mycobacterium* infection. An analysis of the *M. tuberculosis* genome showed the presence of several cyclases and cNMP binding proteins (Cole *et al.*, 1998; McCue *et al.*, 2000) and recently at least two gene products that code for adenylyl cyclases from *M. tuberculosis* have been the focus

Copyright © 2004 John Wiley & Sons, Ltd. 

of biochemical studies (Guo *et al.*, 2001; Reddy *et al.*, 2001; Linder *et al.*, 2002; Shenoy *et al.*, 2003). However, no comprehensive analysis of the genomes from these bacteria has been performed to date in terms of the domain fusions and critical catalytic residues present in these proteins.

In this study, we have analysed the completed genome sequences of members of the Actinobacteria for the presence of nucleotide cyclases represented by all the six classes of nucleotide cyclases. Interestingly, only sequences similar to the class III cyclases are present in these bacteria, and prominent pathogens such as *M. tuberculosis* and *M. leprae* have more than one class III cyclase encoded in their genomes. Many of the putative cyclase genes have interesting domain fusions that have not been identified in other class III cyclases to date, suggesting that biochemical analysis of these proteins and their regulation could be a fruitful area of research in the future.

### Materials and methods

### Dataset

The completed genome sequences of *Mycobacterium tuberculosis* H37Rv (Cole *et al.*, 1998), *M. tuberculosis* CDC1551 (Fleischmann *et al.*, 2002), *M. leprae* (Cole *et al.*, 2001), *M. bovis* AF2122/97 (Garnier *et al.*, 2003), *Streptomyces coelicolor* A3(2) (Bentley *et al.*, 2002), *S. avermitilis* MA-4680 (Omura *et al.*, 2001), *Tropheryma whipplei* str. *twist* (Raoult D, Audic S, Robert C *et al.* 2002. *Tropheryma whipplei* illustrates the diversity of gene loss patterns in small genome bacterial pathogens; unpublished MS), *T. whipplei* TW08/27 (Bentley *et al.*, 2003), *Bifidobacter longum* NCC2705 (Schell *et al.*, 2002), *Corynebacterium glutamicum* ATCC 13 032 (Nakagawa S. 2002. Complete genomic sequence of *Corynebacterium glutamicum* ATCC 13 032; unpublished MS) and *C. efficiens* YS-314 (Kawarabayasi Y, Yamazaki J, Hino Y *et al.* 2002. The entire genomic sequence of *Corynebacterium efficiens* YS-314; unpublished MS) were downloaded from the NCBI website (NCBI **ftp://ftp.ncbi.nih.gov/genomes/Bacteria/**). The combined dataset, consisting of proteins predicted in the genomes, was analysed using the search methods described below. The nrdb was searched on the NCBI website (NCBI **http://www.ncbi.nlm.nih.gov/BLAST/**) for additional actinobacterial sequences from organisms other than those mentioned above. These sequences included completed and unfinished whole genomes and plasmids from these organisms. At the time of analysis (May 2003), 67 different genomes were in various stages of sequencing amongst the Actinobacteria. The preliminary sequence information of the *M. avium* genome was made available from the Institute for Genomic Research (TIGR **http://www.tigr.org/tigr-scripts/ufmg/ReleaseDate.pl**).

### Searches

PSI–BLAST (Altschul *et al.*, 1997) was performed with an inclusion (h) value cut-off of $10^{-4}$ till convergence, using catalytic domain sequences of representative adenylyl and guanylyl cyclases as queries. The catalytic domain of the *M. tuberculosis* Rv1625c gene, shown to have adenylyl cyclase activity (Guo *et al.*, 2001; Reddy *et al.*, 2001), was also used as the seed sequence in PSI–BLAST using amino acids 212–443 (Shenoy *et al.*, 2003). This performed better than other cyclase domain sequences. A position-specific scoring matrix (PSSM) generated using the alignment of the cyclase domains of the 16 *M. tuberculosis* cyclases was also used in the searches. The large number of pseudogenes in *M. leprae* (Cole *et al.*, 2001) prompted us to search for the possible existence of cyclase pseudogenes. This was carried out using the same PSSM in a PSI–TBLASTN search on the nucleotide sequence of the *M. leprae* genome. The amino acid sequences were then mapped on to the annotated genome and all eight of the pseudogenes identified corresponded to genes labelled as pseudogenes earlier (Cole *et al.*, 2001).

Hidden Markov model (HMM) based searches were performed using HMMer (Eddy 2001 **http://hmmer.wustl.edu/**) in the global (ls) and local (fs) mode using the class III cyclase HMM (Accession No. PF00211) from the Protein Families (Pfam) database of alignments and HMMs (Pfam **http://www.sanger.ac.uk/Software/Pfam/**; Bateman *et al.*, 2002) at an Expect (E) value cut-off of 0.1. Additional cyclase sequences from bacteria and eukaryotes were introduced or removed to generate additional models for the searches. The cyclase domains of the 16 *M. tuberculosis* cyclases

Copyright © 2004 John Wiley & Sons, Ltd.

were also used to generate a model for HMM search.

Domain organizations of the cyclases were predicted using the HMMs from the Pfam (Bateman *et al.*, 2002) and the Simple Modular Architecture Research Tool (SMART) websites (SMART **http://smart.embl-heidelberg.de/**; Letunic *et al.*, 2002) and the graphical outputs with modifications have been used in the figures. RPS–BLAST (Schaffer *et al.*, 1999) using the Cluster of Orthologous Groups (Tatusov *et al.*, 2001), Pfam and SMART matrices (NCBI; **ftp://ftp.ncbi.nih.gov/pub/mmdb/cdd/**) was also used along with Conserved Domain Architecture Retrieval Tool (CD ART; **http://www.ncbi.nlm.nih.gov/Structure/lexington/lexington.cgi?cmd=rps**) for assessing domain organization of the sequences. Transmembrane spanning helices were predicted using TMHMM (**http://www.cbs.dtu.dk/services/TMHMM/**). Multiple sequence alignments were computed using HMMalign (Eddy, 2001; **http://hmmer.wustl.edu/**), ClutalX (Jeanmougin *et al.*, 1998) and T-Coffee (Notredame *et al.*, 2000) and were subsequently manually edited. Clustal X was used to generate Figures 2 and 5. Alignments of the cyclase domains were then transferred to the Molecular Evolutionary Genetics Analysis software (Kumar *et al.*, 2001) for generating and bootstrapping the neighbour-joining tree.

### Results

### Cyclases in Actinobacteria

The presence of multiple classes of cyclases, without sequence similarity to each other, indicates the independent evolution of proteins for catalysing the conversion of ATP to cAMP. PSI–BLAST searches with query sequences of the all classes of adenylyl cyclases in Actinobacteria yielded hits above the E value cut-off defined in Materials and methods, for only the class III cyclases. PSI–BLAST and HMM searches (see Materials and methods) for sequences similar to the currently known class III cyclases revealed the presence of several class III cyclase members within the genomes of many Actinobacteria (Tables 1–3). We identified 62 proteins in this study, each having a single class III cyclase domain. The genomes of *Bifidobacter longum* and *Tropheryma whipplei* did not reveal the presence of members of any of the currently described classes of nucleotide cyclases,

**Table 1.** Cyclases found in *M. tuberculosis* strains H37Rv and CDC1551 and *M. bovis*

| | Gene names | Gene identifiers | *M. tb* CDC1551 gene | *M. bovis* gene | Protein length (aa) |
|---|---|---|---|---|---|
| Soluble cyclases | Rv0891c | gi 1314030 | MT0915 | Mb0915c | 285 |
| | Rv1120c | gi 2117214 | MT1152 | Mb1151c | 164 |
| | Rv1264 | gi 480322 | MT1302 | Mb1295 | 397 |
| | Rv1647 | gi 1838999 | MT1685 | Mb1674 | 328 |
| | Rv1359 | gi 1419062 | MT1403 | Mb1394 | 253 |
| | Rv1900c | gi 2225960 | MT1951 | Mb1935c | 462 |
| | Rv2212 | gi 1237065 | MT2268 | Mb2235 | 388 |
| Membrane-bound cyclases | Rv1625c | gi 2113909 | MT1551 | Mb1651c | 443 |
| | Rv1318c | gi 1340084 | MT1359 | Mb1352c | 541 |
| | Rv1319c | gi 1340085 | MT1361 | Mb1353c | 535 |
| | Rv1320c | gi 1340086 | MT1362 | Mb1354c | 567 |
| | Rv3645 | gi 2105049 | MT3748 | Mb3669 | 549 |
| | Rv2435c | gi 1666149 | MT2509 | Mb2461c | 730 |
| Multidomain DNA-binding cyclases | Rv0386 | gi 2909507 | MT0399 | Mb0393 | 1092 |
| | Rv1358 | gi 1419061 | MT1402 | Mb1393 | 1159 |
| | Rv2488c | gi 2791528 | MT2563 | Mb2515c | 1199 |

The genome of *M. bovis* is highly similar to that of *M. tuberculosis* and also has identical cyclase genes. The primary annotations for the *M. tuberculosis* CDC1551 and *M. bovis* genomes for corresponding genes from *M. tuberculosis* H37Rv are listed. The genes are classified based on the domain analysis that has been performed and described in this study. The protein lengths in amino acids (aa) for the *M. tuberculosis* H37Rv proteins are given, as per the primary annotation. *M. tuberculosis* CDC1551 has one cyclase more than the other two mycobacteria (MT1360).

Copyright © 2004 John Wiley & Sons, Ltd. 

**Table 2.** Cyclases identified in *M. leprae*

| Status | Gene identifiers | Gene names | Protein length (aa) | *M. tb* orthologue |
|---|---|---|---|---|
| Full-length genes | gi 13093284 | ML1399 | 324 | Rv1647 |
| | gi 13093492 | ML1753 | 1106 | Rv1358 |
| | gi 13093954 | ML2341 | 732 | – |
| | gi 13092553 | ML0201 | 530 | Rv3645 |
| | *M. leprae* gene | *M. tb* orthologue | | |
| | ML0465 | Rv1320c | | |
| | ML1111 | Rv1264 | | |
| | ML1154 | Rv1318c | | |
| Pseudogenes | ML1242 | Rv0386 | | |
| | ML1285 | Rv1625c | | |
| | ML1820 | Rv1319c | | |
| | ML1948 | Rv3645 | | |
| | ML2016 | Rv1900c | | |

The full-length genes (gene identifiers and primary annotations are listed) represent those that code for proteins that match genes in *M. tuberculosis (M. tb)*. The ML2341 cyclase is unique to *M. leprae*. The pseudogenes corresponding to cyclases were identified by PSI–TBLASTN and have been identified in the original genome annotation (Cole *et al.*, 2001). The protein length is given in amino acids (aa).

**Table 3.** Cyclases identified in this study in various members of the Actinobacteria

| Organisms | Gene identifiers | Gene names | Protein length (aa) |
|---|---|---|---|
| *Corynebacterium efficiens* YS-314 | gi 25026866 | CE0310 | 538 |
| *Corynebacterium glutamicum* ATCC 13032 | gi 19551561 | Cgl0311 | 486 |
| *Streptomyces coelicolor* A3(2) | gi 21223302 | SCK13.20 | 381 |
| *Streptomyces avermitilis* MA-4680 | gi 29829871 | SAV3328 | 405 |
| *Streptomyces griseus* | gi 11131886 | | 399 |
| *Brevibacterium liquifaciens* | gi 117790 | | 403 |
| *Thermobifida fusca* | gi 23018870 | | 358 |
| *Arthrobacter nicotinovorans* | gi 25169176 | | 323 |
| *Actinosynnema pretiosum* subsp. *auranticum* | gi 21449364 | | 913 |

Gene identifiers and primary annotations for completed genome sequences are shown. The protein lengths are given in amino acids (aa).

and therefore these organisms may have no need of cAMP as a regulatory molecule, or have novel proteins that need to be identified through classical screening procedures.

### Cyclases in *M. tuberculosis* and *M. bovis*

An analysis of the *M. tuberculosis* H37Rv genome for the presence of cNMP binding proteins and cNMP metabolizing enzymes has been reported earlier, and predicted 15 cyclases in the genome (McCue *et al.*, 2000). Recently, the genome sequence of the CDC1551 strain has been made available (Fleischmann *et al.*, 2002) and we have analysed this genome as well for the members of the nucleotide cyclase family, using methods described above. Searches with queries from families other than the class III cyclase family did not yield hits with scores above cut-off, as described in Materials and methods. The largest number (17) of cyclases is seen in the genome of *M. tuberculosis* CDC1551, followed by the *M. tuberculosis* H37Rv genome which, in our analysis, shows 16 putative cyclase genes (Table 1). The presence of one extra cyclase in the CDC1551 strain compared to the H37Rv strain is explained by expansion of the Rv1318c cyclase gene family (Fleischmann *et al.*, 2002). All *M. tuberculosis* putative cyclase genes have only a single class III cyclase domain per polypeptide chain, in contrast

Copyright © 2004 John Wiley & Sons, Ltd. 

to the eukaryotic adenylyl cyclases. Highly similar genes (>99% sequence identity) are present in the CDC1551 strain (the gene names from the CDC1551 strain are mentioned in parentheses in the text). A variety of domains are found fused to the cyclase domain in these 16 genes (Figure 1) and some of these fusions are unique to the actinobacterial cyclases (see below). The genome of *M. bovis* reveals the presence of 16 cyclases identical to those in *M. tuberculosis* H37Rv (Table 1).

### Genes that code for proteins with only an identifiable cyclase domain

There are six genes in the *M. tuberculosis* genome that, upon prediction, seem to code for only a class III cyclase domain, with no additional domains identifiable from current databases. These are Rv0891c (MT0915), Rv1120c (MT1152), Rv1264 (MT1302), Rv1359 (MT1403), Rv1647 (MT1685), and Rv2212 (MT2268) (see Table 1, Figure 1). As shown in Table 1, Rv0891c and Rv1359 contain the number of amino acids minimally required for a functional cyclase domain. Rv1264, Rv1647 and Rv2212 are longer proteins and contain >100 amino acids adjacent to the cyclase domain. However, these sequences do not contain additional domains that are listed in Pfam currently.

Rv0891c appears to have all the residues required for catalytic activity in class III cyclases. However, it has non-conserved residues (arginine and leucine) at positions responsible for substrate selectivity, in contrast to those seen in the mammalian enzymes (Figure 2). A clustering based on the sequence alignment of representative actinobacterial cyclase domains identified in this study and other class III cyclases (Figure 3) shows that the Rv0891c cyclase domain is more related to the fungal and parasite cyclases and a group of Actinobacteria-specific NB-ARC (nucleotide-binding–common to Apaf1, plant resistance gene products and CED4) domain (van der Biezen *et al.*, 1998) containing cyclases as described below.

The Rv0891c (MT0915) gene is adjacent to the Rv0890c gene which is predicted to have a NB-ARC domain and a C-terminal helix-turn-helix (HTH) DNA binding domain and the operonic nature of these two genes (TIGR **http://www.tigr.org/tigr-scripts/operons/pairs.cgi?taxon_id=89**) hints at their possible functional interplay.

A close examination of Rv1120c (MT1152) reveals that the protein is only 164 amino acids long, which is smaller than a typical class III cyclase catalytic domain (∼200–250 amino acids). A number of conserved class III cyclase-like residues are present in Rv1120c, but it lacks the more C-terminal residues responsible for substrate selectivity and transition-state stabilization (Figure 2). A BLASTN search on the *M. avium* genome reveals the presence of an orthologue (80% identity to the first 149 amino acids of Rv1120c) whose protein length is long enough to encode a complete cyclase domain. We therefore aligned the nucleotide sequence in the H37Rv genome, beyond the predicted stop codon of the Rv1120c, against the corresponding region of the unfinished genome of *M. avium* and found that a single base deletion in the *M. tuberculosis* genome has led to a frame shift and premature truncation of the Rv1120c polypeptide (Figure 4). The putative Rv1120c orthologue in *M. avium* extends to the end of an ORF that is contained within the Rv1119c gene in *M. tuberculosis.* Therefore, the putative gene in *M. avium* possesses functional class III cyclase C-terminal residues that are lacking in the *M. tuberculosis* protein. It is possible therefore, that a loss of the full-length cyclase, seen in the Rv1120c cyclase gene in *M. tuberculosis* H37Rv (and CDC1551), has occurred and that Rv1120c could represent the only cyclase pseudogene in *M. tuberculosis.* The status of the Rv1120c orthologue in *M. bovis* is identical to that in *M. tuberculosis*.

The Rv1264 (MT1302) adenylyl cyclase was recently characterized biochemically and its N-terminal region was found to downregulate the activity of the catalytic domain (Linder *et al.* 2002). Rv1264c is more closely related to the *Streptomyces* cyclases in having a short deletion just N-terminal to the substrate selective aspartate residue (Figure 2). Another related cyclase is the Rv2212 (MT2268) cyclase, which is as yet uncharacterized biochemically. The clustering shown in Figure 3 reveals that it positions in a group of cyclases that includes proteins from *Streptomyces, Brevibacterium* and *Thermobifida.* Rv2212 has all the residues required for catalytic activity and appears to be specific for ATP as a substrate (Figure 2). Rv1264 has sequence similarity (29% identity in the cyclase domain) to the cyclase from *Brevibacterium*, which has been shown to be activated by pyruvate. However, Rv1264 was not stimulated by

Copyright © 2004 John Wiley & Sons, Ltd. 

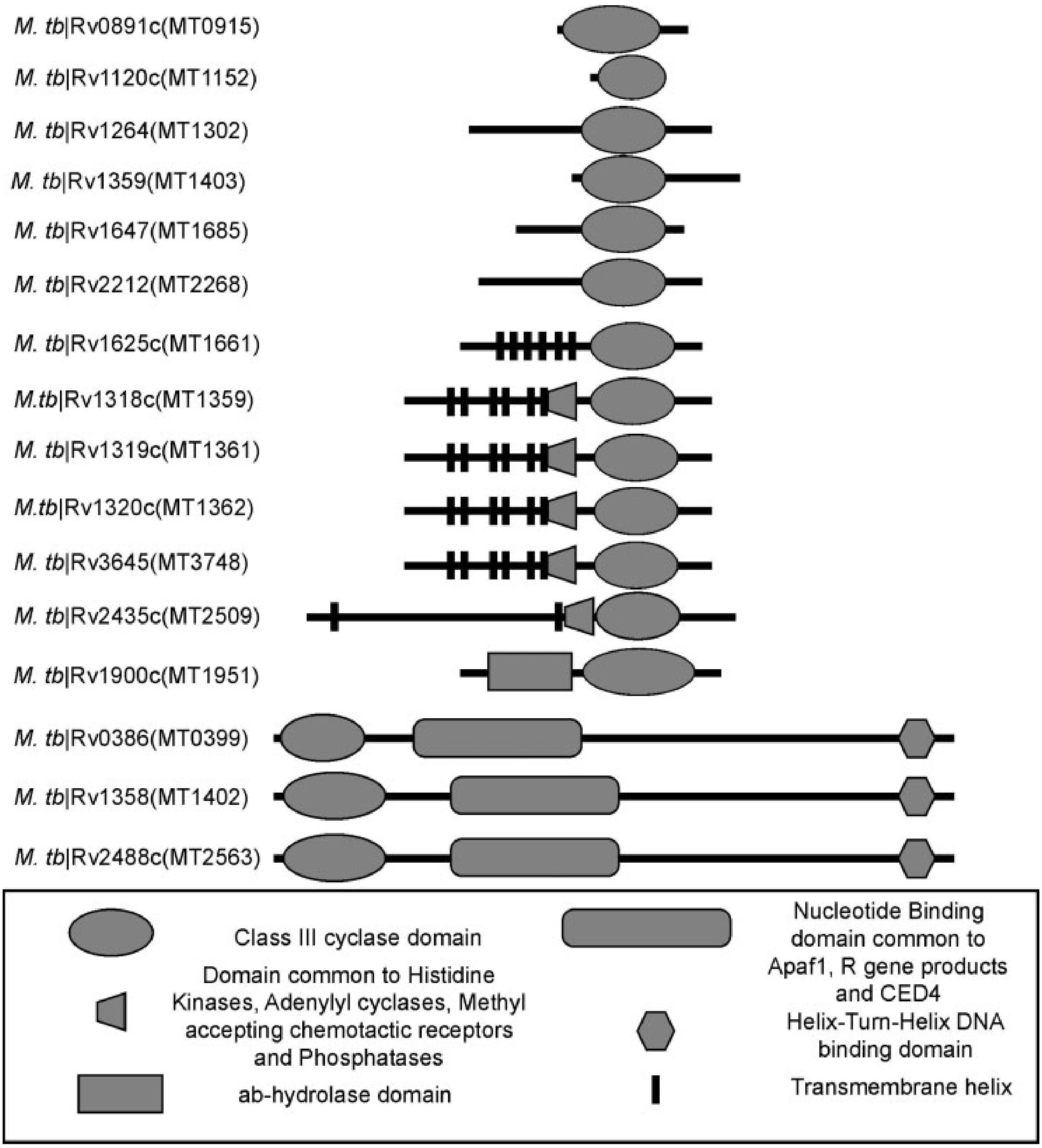

**Figure 1.** Domain organizations of the *M. tuberculosis* cyclases. Schematic domain organizations drawn approximately to scale are shown for the 16 proteins identified in the *M. tuberculosis* H37Rv strain. The corresponding *M. tuberculosis* CDC1551 gene names are given in parentheses. It is interesting to note that *M. tuberculosis* has four cyclases (MT1359, MT1360, MT1361, MT1362) similar to three cyclases found in *M. tuberculosis* H37Rv (Rv1318c, Rv1319c, Rv1320c)

pyruvate or other small molecules (Linder *et al.*, 2002).

Both Rv1359 (MT1403) and the adjacent Rv1358 (MT1402) genes contain the class III cyclase domain. However, Rv1359 has higher sequence similarity to the Rv0386 (MT0399) cyclase than to its neighbour (47% identity to Rv0386 compared to 38% to Rv1358). In fact, Rv0386 is an enzyme

Copyright © 2004 John Wiley & Sons, Ltd. 

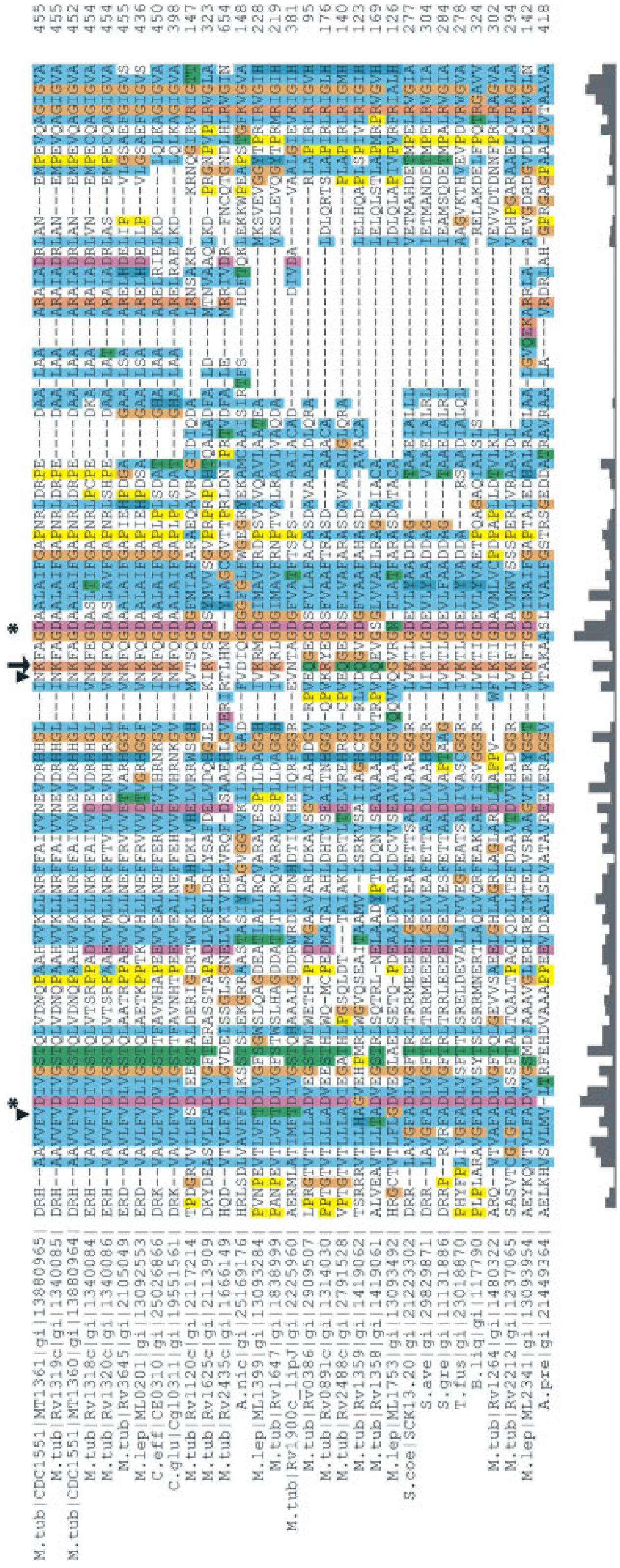

Copyright © 2004 John Wiley & Sons, Ltd. 

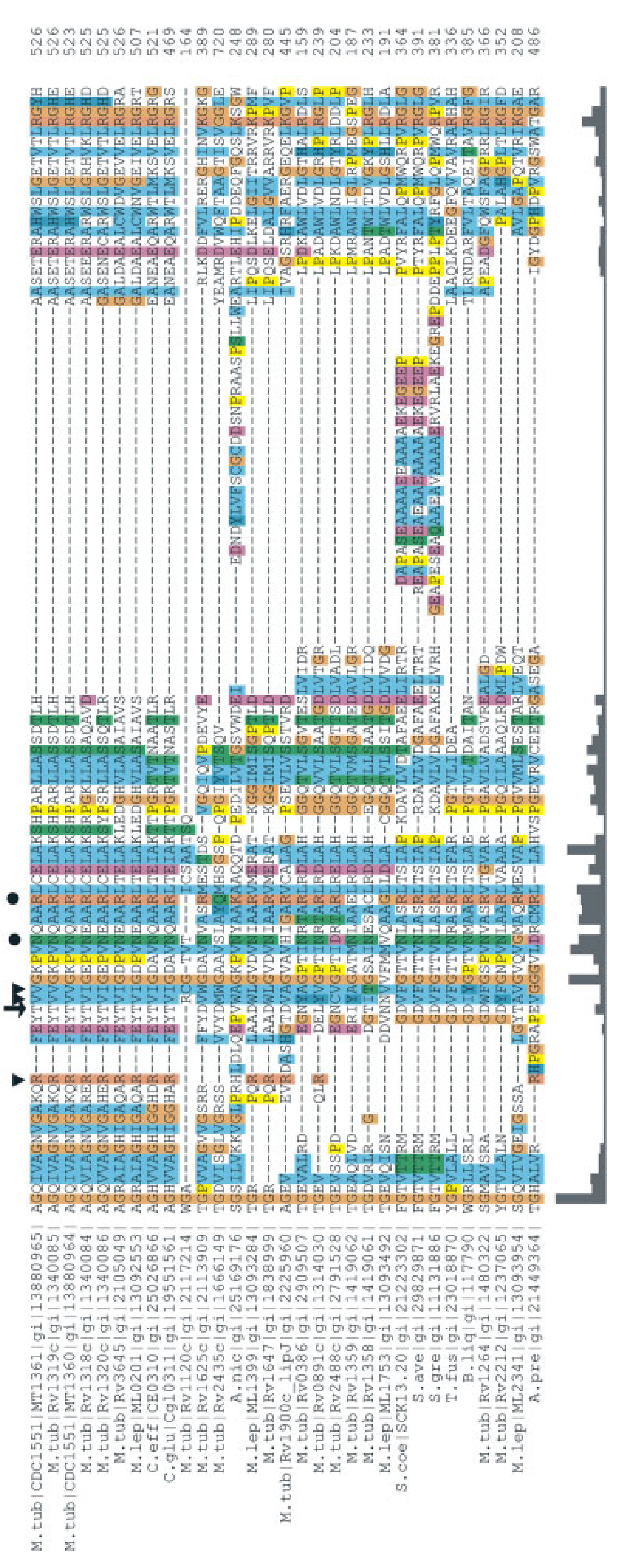

**Figure 2.** Multiple sequence alignment of actinobacterial cyclase domains. Full-length protein sequences were aligned to the class III cyclase HMM profile using HMMalign and then realigned with T-Coffee and manually edited. A.nic, *A. nicotinovorans*; A.pre, *A. pretiosum*; B.liq, *B. liquefaciens*; C.eff, *C. efficiens*; C.glu, *C. glutamicum*; M.tub, *M. tuberculosis*; M.lep, *M. leprae*; S.ave, *S. avermitilis*; S.coe, *S. coelicolor*; S.gre, *S. griseus*; T.fus, *T. fusca*. The gene names as per the genome annotations, along with gene identifiers, have been used. The metal binding aspartates (*), substrate selectivity residues identified biochemically (↓), a computational approach (▼) and the transition state stabilization residues (●) are indicated

Copyright © 2004 John Wiley & Sons, Ltd.

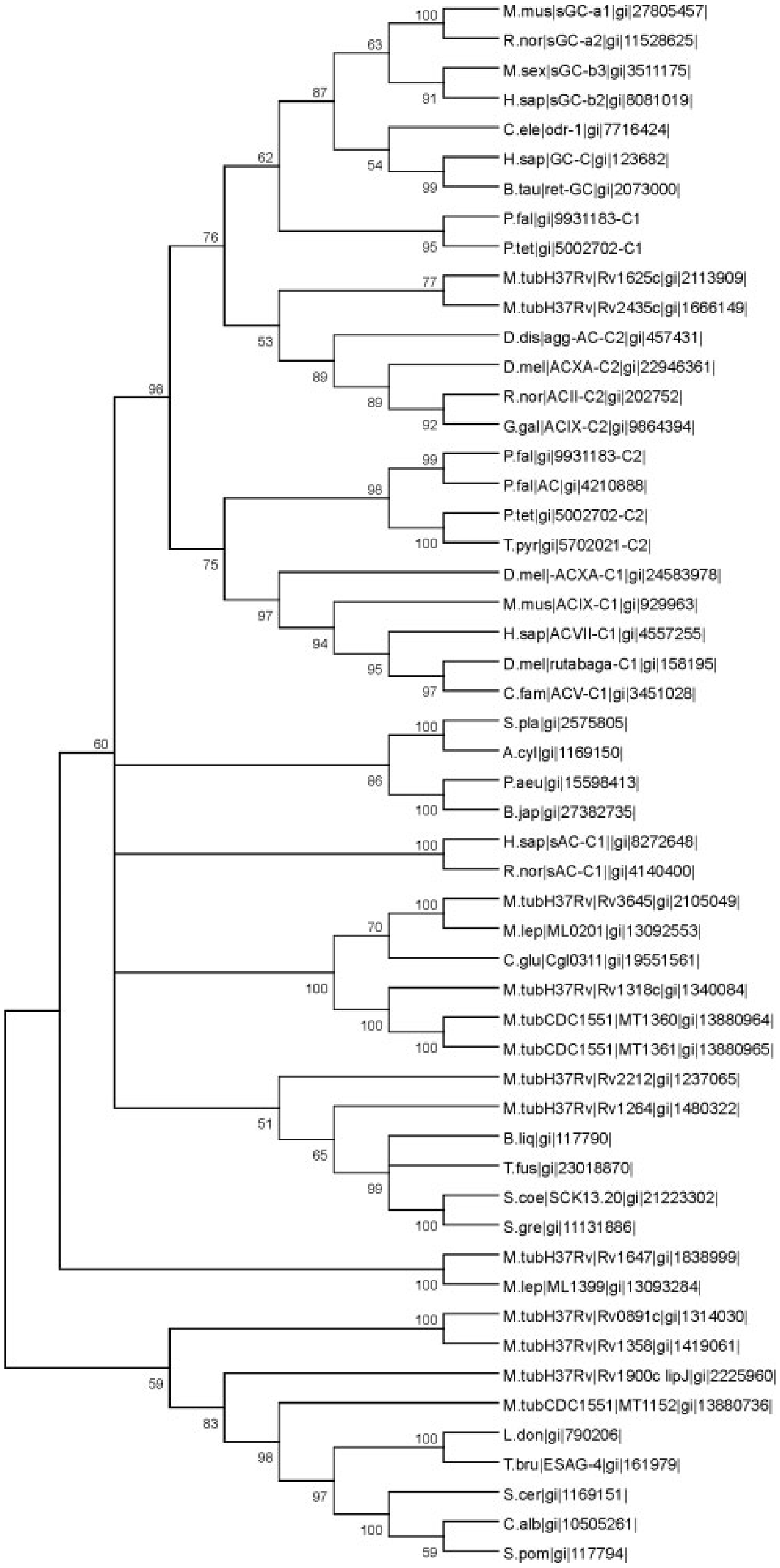

M.mus|sGC-a1|gi|27805457|

R.nor|sGC-a2|gi|11528625|

M.sex|sGC-b3|gi|3511175|

H.sap|sGC-b2|gi|8081019|

C.ele|odr-1|gi|7716424|

H.sap|GC-C|gi|123682|

B.tau|ret-GC|gi|2073000|

P.fal|gi|9931183-C1

P.tet|gi|5002702-C1

M.tubH37Rv|Rv1625c|gi|2113909|

M.tubH37Rv|Rv2435c|gi|1666149|

D.dis|agg-AC-C2|gi|457431|

D.mel|ACXA-C2|gi|22946361|

R.nor|ACII-C2|gi|202752|

G.gal|ACIX-C2|gi|9864394|

P.fal|gi|9931183-C2|

P.fal|AC|gi|4210888|

P.tet|gi|5002702-C2|

T.pyr|gi|5702021-C2|

D.mel|-ACXA-C1|gi|24583978|

M.mus|ACIX-C1|gi|929963|

H.sap|ACVII-C1|gi|4557255|

D.mel|rutabaga-C1|gi|158195|

C.fam|ACV-C1|gi|3451028|

S.pla|gi|2575805|

A.cyl|gi|1169150|

P.aeu|gi|15598413|

B.jap|gi|27382735|

H.sap|sAC-C1||gi|8272648|

R.nor|sAC-C1||gi|4140400|

M.tubH37Rv|Rv3645|gi|2105049|

M.lep|ML0201|gi|13092553|

C.glu|Cgl0311|gi|19551561|

M.tubH37Rv|Rv1318c|gi|1340084|

M.tubCDC1551|MT1360|gi|13880964|

M.tubCDC1551|MT1361|gi|13880965|

M.tubH37Rv|Rv2212|gi|1237065|

M.tubH37Rv|Rv1264|gi|1480322|

B.liq|gi|117790|

T.fus|gi|23018870|

S.coe|SCK13.20|gi|21223302|

S.gre|gi|11131886|

M.tubH37Rv|Rv1647|gi|1838999|

M.lep|ML1399|gi|13093284|

M.tubH37Rv|Rv0891c|gi|1314030|

M.tubH37Rv|Rv1358|gi|1419061|

M.tubH37Rv|Rv1900c lipJ|gi|2225960|

M.tubCDC1551|MT1152|gi|13880736|

L.don|gi|790206|

T.bru|ESAG-4|gi|161979|

S.cer|gi|1169151|

C.alb|gi|10505261|

S.pom|gi|117794|

that as far as domain organization is concerned, is identical to Rv1358 (Figure 1 and see below). With the absence of one of the metal binding aspartates as well as the critical arginine in its gene product, Rv1359 is likely to be inactive as a homodimer (Figure 2).

The catalytic domain of Rv1647 (MT1685) is similar (28–34% sequence identity) to several cyclases from nitrogen-fixing soil bacteria such as *Sinorhizobium meliloti* and *Mesorhizobium loti*. The gene appears to harbour the residues required for catalytic activity and therefore is likely to possess cyclase activity (Figure 2). The same region of the protein also picks up the GGDEF domain (Galperin *et al*., 2001) when the SMART database PSSM profiles are used during RPS–BLAST (E value 0.01). The GGDEF domain is thought to be homologous to the class III cyclase domain. However, with a very robust hit (E value $10^{-28}$) to the class III cyclase domain, and the presence of all residues required for catalysis, it is probable that its gene product could be an active adenylyl cyclase.

### Cyclases with additional non-cyclase domain fusions

### Membrane-bound cyclases

Several putative cyclases in the *M. tuberculosis* genome have transmembrane helices and hence could be localized to the membrane (Figure 1). There are six predicted membrane-bound cyclases in the H37Rv genome and seven in the CDC1551 genome (accounted for by the addition to the Rv1318c family). Interestingly, a majority of these putative cyclases contain six transmembrane spanning domains. In addition, except Rv1625c and its orthologues in *M. tuberculosis* CDC1551 (MT1661) and *M. bovis* (Mb1651c), all the other putative membrane-associated cyclases have an intracellular juxtamembrane HAMP (present in histidine kinases, adenylyl cyclases, methyl accepting chemotactic receptors and phosphatases) domain (Aravind *et al*., 1999b, Gālperin *et al*., 2001), followed by the C-terminal cyclase domain.

The Rv1625c adenylyl cyclase with six transmembrane helices and a single catalytic domain is the adenylyl cyclase gene product that has been the most studied to date (Guo *et al*., 2001; Reddy *et al*., 2001; Shenoy *et al*., 2003). Homodimerization of the Rv1625c gene product is seen, and would give rise to a 12-transmembrane-spanning domain containing enzyme that is similar to the mammalian enzymes.

The high sequence similarity of the Rv1625c gene product with the mammalian enzymes (35–40% identity to eukaryotic adenylyl and guanylyl cyclases) does not, however, translate into identity in its biochemical behaviour (Shenoy *et al*., 2003). The protein could not be converted into a guanylyl cyclase by replacement of ATP-specifying residues to those for GTP present in guanylyl cyclases, probably due to alterations in the dimeric status and improper juxtaposition of the critical interfacial residues (Shenoy *et al*., 2003). This indicates a difference in the dimer interface of a homodimeric cyclase such as Rv1625c and that of the heterodimeric mammalian adenylyl cyclases.

The most common domain found to occur in fusion with the cyclase domain in the *M. tuberculosis* strains is the HAMP domain (Figure 1). The HAMP domain is believed to act as a structural linker between a sensor domain and the C-terminal

**Figure 3.** Clustering of representative actinobacterial cyclases. Cyclase domains of representative actinobacterial and eukaryotic, bacterial and fungal cyclases were multiply aligned using ClustalX and a neighbour-joining tree was drawn using MEGA. The bootstrapped condensed tree with a cut-off of 50% is shown with values mentioned on the nodes. Notice the presence of *M. tuberculosis* cyclases within groups of eukaryotic C2 domains and fungal/parasite cyclase clusters and the *Streptomyces/Brevibacterium* cyclase clusters. A.cyl, *Anabaena cylindrica*; B.jap, *Bradyrhizobium* japonicum; B.liq, *Brevibacterium liquefaciens*; B.tau, *Bos taurus*; C.alb, *Candida albicans*; C.ele, *Caenorhabditis* elegans; C.fam, *Canis familiaris*; C.glu, *Corynebacterium glutamicum*; D.dis, *Dictyostelium discoideum*; D.mel, *Drosophila melanogaster*; G.gal, *Gallus gallus*; H.sap, *Homo sapiens*; L.don, *Leishmania donovanii*; M.lep, *M. leprae*; M.mus, *Mus musculus*; M.sex, *Manduca sexta*; M.tub, *Mycobacterium tuberculosis*; P.aeu, *Pseudomonas aeruginosa*; P.fal, *Plasmodium falciparum*; P.tet, *Paramaecium tetraauerelia*; R.nor, *Rattus norvegicus*; S.cer, *Saccharomyces cerevisiae*; S.coe, *Streptomyces coelicolor*; S.gre, *S. griseus*; S.pla, *Sprirulina platensis*; S.pom, *Schizosaccharomyces pombe*; T.bru, *Trypanosoma brucei*; T.fus, *Thermobifida fusca*; T.pyr, *Tetrahymena pyriformis*. Actinobacterial cyclases are named with the primary annotations and gene identifiers. Gene identifiers for all other proteins are given. C1 and C2 stand for the first and second class III cyclase domains found in eukaryotic membrane-bound adenylyl and parasitic guanylyl cyclases. Guanylyl cyclases are named as GC and the soluble guanylyl cyclase subunits are named as a and b. Adenylyl cyclases are named as AC and the isoforms are given in Roman numerals

Copyright © 2004 John Wiley & Sons, Ltd. 

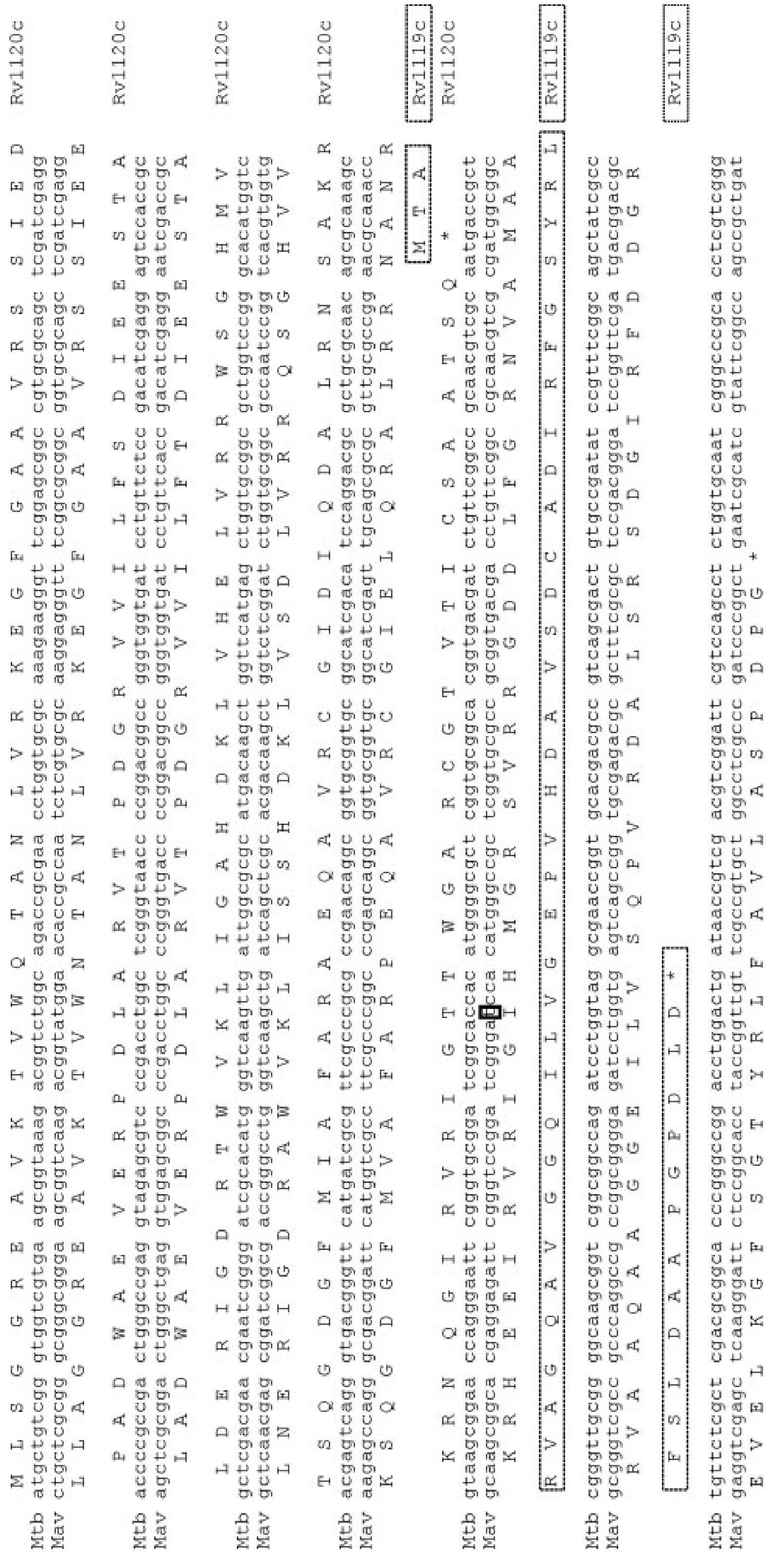

**Figure 4.** Identification of the Rv1120c gene as a cyclase pseudogene. Nucleotide sequence of the annotated Rv1120c gene from *M. tuberculosis* (Mtb) is shown with the amino acid translation above the sequence. The gene names are indicated on the right. The sequence of the corresponding gene from *M. avium* (Mav) is shown with the translation depicted below the sequence. The single base deleted in *M. tuberculosis* that gives rise to a frame shift and premature truncation of the Rv1120c gene product, is shown in a black box on the *M. avium* sequence. The gene annotated as Rv1119c is shown within a dotted box. Notice the high sequence similarity between the *M. tuberculosis* and *M. avium* sequences at the N-terminus of Rv1120, and the C-terminal sequence of Rv1119c to the C-terminus of the *M. avium* protein effecter domain, e.g. the kinase, phosphatase or, as in the study here, a cyclase domain (Galperin *et al.*, 2001). Mutations in the HAMP domain have been usually known to cause constitutive activation of fused downstream effecter domains (Appleman *et al.*, 2003). The conserved proline and glutamate residues conserved in HAMP domains are also conserved in the HAMP domains of actinobacterial cyclases (Figure 5A). There are four members of this type of cyclase in the H37Rv strain and five in CDC1551 (Fleischmann *et al.*, 2002), where the six transmembrane helices are followed by a HAMP domain. Expression of one of these representatives, the Rv1320c cyclase, in *E. coli* led to the production of protein that was inactive (Reddy, *et al.* 2001). However, sequence alignment reveals that Rv1320c, Rv1319c and Rv1318c all have residues required for catalytic activity (Figure 2), and it is anticipated that the catalytic domains alone of these proteins may have adenylyl cyclase activity, with the HAMP domain providing a regulatory role. The likelihood that this family of proteins could be important for the physiology of the organism is suggested by the fact that the Rv1319c transcript has been detected by microarray analysis as being repressed during hypoxia (Sherman *et al.*, 2001).

The Rv3645 (MT3763) cyclase also has six transmembrane helices similar to the Rv1318c family of proteins, and all residues required for catalysis are present in the catalytic domain (Figures 1, 2). Interestingly, as described later, *M. leprae* and *Corynebacterium* have orthologues of this cyclase rather than those from the Rv1318 cyclase family. A multiple sequence alignment of the HAMP domain of Rv3645 with other HAMP domains is shown in Figure 5A.

The Rv2435c (MT2509) cyclase has two transmembrane domains and intracellular HAMP and cyclase domains (Figure 1), and therefore has similarity in domain organization with the two-transmembrane bacterial chemotaxis receptors (Mowbray *et al.*, 1998). This gene product could therefore represent a receptor that senses some unknown ligand. Its N-terminal region up to the catalytic domain is similar to a methyl-accepting chemotaxis protein from *Vibrio vulnificus* (27% identity to gi 27 366 778 with an E value of $4 \times 10^{-43}$). The similarity extends within the HAMP domain as well and it appears that the fusion of this module to a cyclase domain gave rise to this protein in mycobacteria. However, the HAMP domain in Rv2435c differs significantly in sequence from the HAMP domain in the Rv1318c family (Figure 5A), suggesting that the event that brought together the HAMP domain and a cyclase domain in a single protein occurred independently more than once during the evolution of the *M. tuberculosis* genome.

At the nucleotide level, the stop codon of Rv2435c overlaps by one base with the start codon of Rv2434c, suggesting that the Rv2425c cyclase exists in an operon with Rv2434c (TIGR; **http://www.tigr.org/tigr-scripts/operons/pairs.cgi?taxon_id=89**). The latter gene is a transmembrane channel with a C-terminal cNMP-binding domain (Cole *et al.*, 1998; McCue *et al.*; 2000) and might thus be functionally regulated by Rv2435c. One report suggests that the Rv2435c and Rv2434c transcripts are repressed during hypoxia (Sherman *et al.*, 2001). Despite this interesting insight, it is puzzling to note that Rv2435c lacks one of the metal binding sites and both the transition state-stabilizing asparagine and arginine residues (Figure 2).

### Soluble cyclases

There are four cyclases Rv0386 (MT0399), Rv1358 (MT1402), Rv2488c (MT2563) and Rv1900c (MT1951) in the *M. tuberculosis* genomes that do not appear to contain any transmembrane helices, and therefore could be cytosolic enzymes (Table 1, Figure 1).

The Rv1900c (MT1951) is annotated as lipJ (Cole *et al.*, 1998) and has an N-terminal lipid esterase domain that is identified by PSI–BLAST (Figures 1, 5B). The corresponding gene in the *M. bovis* genome is annotated as a probable lignin peroxidase (Mb1935c). The N-terminal region of Rv1900c is similar to the 3-oxoadipate enol-lactone hydrolase from *Pseudomonas* sp. (22% identity to gi 17 736 948 at E value $5 \times 10^{-91}$) which is a protein amongst the top hits that has been biochemically characterized (Gobel *et al.*, 2002). This domain is also identified as a $\alpha\beta$-hydrolase domain identified in the COG database (COG0596, MhpC, predicted hydrolase or acyltransferase). The C-terminal region of Rv1900c is similar to class III cyclases (Figure 2) and therefore this unique domain combination makes it interesting to investigate whether both domains are enzymatically functional. The cyclase domain, however, lacks the critical asparagine residue (Figure 2) and might therefore play only a regulatory role, by providing a site

Copyright © 2004 John Wiley & Sons, Ltd. 

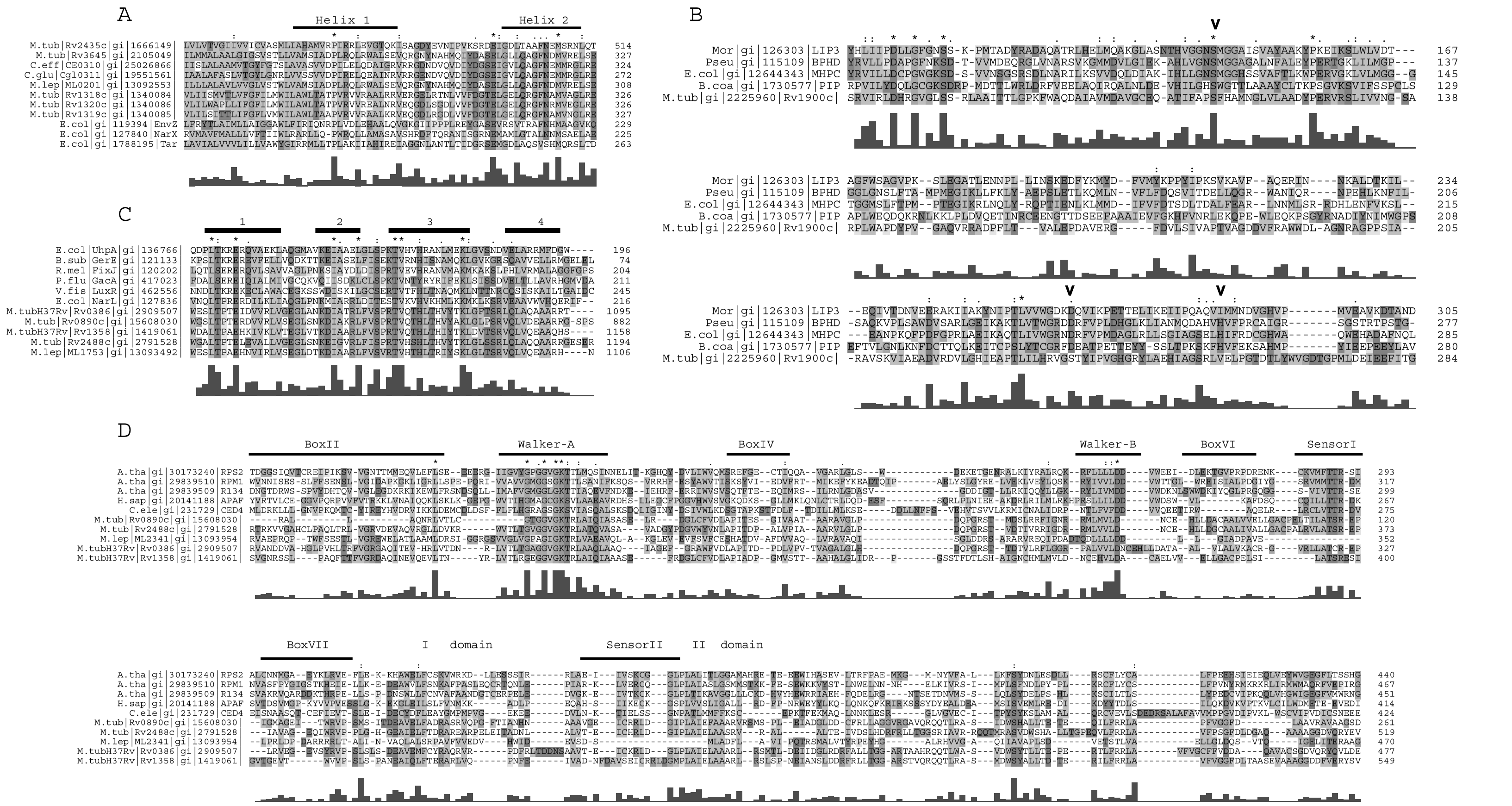

**Figure 5.** Alignment of non-cyclase domains found in actinobacterial cyclases. (A) Alignment of the HAMP domain found in the cyclases identified here is shown along with three proteins (EnvZ, NarX and Tar) that have HAMP domains from *E. coli* (E.col). The HAMP domain is believed to have two amphipathic helices, as shown in the figure, which have been studied by mutagenesis (Appleman *et al.* 2003). (B) Alignment of representative members of the $\alpha\beta$-hydrolase family with the N-terminal region of Rv1900c. The proteins shown in the alignment are triglycerol lipase (lipase 3; LIP3) from *Moraxella* (Mor), 2-hydroxy-6-oxo-6-phenylhexa-2,4-dienoate hydrolase (BPHD) from *Pseudomonas* (Pseu), 2-hydroxy-6-keto-nona-2,4-diene 1,9-dioic acid 5,6-hydrolase (MHPC) from *E. coli* (E.col) and proline iminopeptidase (PIP) from *Bacillus coagulans.* The active site triad are indicated by arrow heads. (C) Alignment of the HTH domains of actinobacterial cyclases and members of the GerE [from *Bacillus subtilis* (B.sub)] family of HTH DNA-binding transcription regulators. UphA from *E. coli* (E.col), FixJ from *Rhizobium meliloti* (R.mel), GacA from *Pseudomonas fluorescens* (P.flu) and NarL from *E. coli* are two-component response regulators, while LuxR from *Vibrio fischeri* (V.fis) is involved in quorum sensing. The numbers 1–4 stand for alpha helices. (D) Alignment of the NB-ARC domain from actinobacterial cyclases and representative proteins. The proteins shown are *Arabidopsis thaliana* (A.thal) resistance proteins, such as resistance to *Pseudomonas syringae* protein 2 (RPS2), resistance to *P. syringae* protein 3 (RPM1) and putative resistance RPP13-like protein 4 (R134). In addition there are *Homo sapiens* (H.sap) Apaf1 and *Caenorhabditis elegans* (C.ele) CED4 proteins. The regions within the NB-ARC domain are labelled as identified earlier (Neuwald *et al.*, 1999, Jaroszewski *et al.*, 2000)

Copyright © 2004 John Wiley & Sons, Ltd.

for binding of an allosteric small molecule such as a nucleotide. An alignment of the N-terminal region of Rv1900c that encompasses the $\alpha\beta$-hydrolase domain reveals the presence of the conserved active site serine residue that is known in other enzymes of this family. However, the other two residues in the catalytic triad (Ollis *et al.*, 1992), an aspartate or glutamate and a histidine are not present (Figure 5B).

Three cyclases (Rv0386, Rv1358 and Rv2488c) are found in fusion with a NB-ARC domain that is C-terminal to the cyclase domain, and are the only proteins with a cyclase domain at the extreme N-terminus. The NB-ARC domain is a nucleotide binding domain of the $AAA^+$ superfamily (ATPases Associated with several cellular Activities), which has Walker motifs required for nucleotide binding (Neuwald *et al.*, 1999). The NB-ARC domain has been shown in some proteins to bind and hydrolyse ATP, thereby acting as a switch that regulates the protein's function. In some proteins ATP hydrolytic activity is absent, and the domain has been shown to be involved in oligomerization and the formation of large protein assemblies (Jaroszewski *et al.*, 2000).

These soluble cyclases also possess an extreme C-terminal LuxR-type HTH domain (Bateman *et al.*, 2002). A similar domain organization is absent outside the mycobacteria, based on an analysis with available genome sequences (Shenoy AR, unpublished observations), and suggests the importance of this family of cyclases in the biology of mycobacteria. Several transcription factors are known to have an ATPase domain and the HTH domain (Yeats *et al.*, 2003), and therefore the presence of a class III cyclase domain N-terminal to these domains is suggestive of an interesting regulation of a possible DNA-binding activity of these proteins. The HTH domain in these cyclases is similar to the GerE/LuxR family of transcription regulators. Figure 5C shows the multiple sequence alignment of HTH domains from the actinobacterial cyclases and other members of this family.

As described earlier, the Rv0891c soluble cyclase exists in an operon with the NB-ARC and HTH-containing Rv0890c protein. The observation that the NB-ARC and HTH-containing domain protein gene is found adjacent to a gene encoding a putative cyclase domain in the genome suggests an interplay between these domains, which may have led to the ultimate evolution of a protein containing all three domains fused together in *M. tuberculosis* species. This is further supported by BLAST searches with the Rv0891c and Rv0890c cyclases that show Rv0386, Rv1358 and Rv2488c cyclases as the top three hits (data not shown). The NB-ARC domains of these proteins show the presence of typical Walker A and B motifs (Figure 5D) identified in the $AAA^+$ superfamily of proteins (Neuwald *et al.*, 1999; Jaroszewski *et al.*, 2000). These domains were also identified earlier in a study for ATPases involved in apoptosis (Aravind *et al.*, 2001).

Amongst the soluble cyclases, only the Rv0386 cyclase seems to possess all residues required for catalysis (Figure 2). The Rv1358 cyclase lacks the second metal binding aspartate as well as the critical asparagine residue. Rv2488c lacks the critical arginine residue (Figure 2). The absence of these residues might indicate the lack of activity, unless there are other compensatory mutations to overcome the effect of these substitutions in the overall structure of these proteins. However, these cyclases do not have any of the typical adenylyl or guanylyl cyclase-like residues at the positions known to select for substrate selectivity. Thus, the nucleotide that these cyclases bind can only be determined through biochemical studies, although it seems more likely that this variant of the class III cyclase might be better suited to bind stretches of DNA sequences, due to the lack of well-defined nucleotide selectivity.

### Cyclases in *Mycobacterium leprae*

The *M. leprae* genome has a large number of pseudogenes (Cole *et al.*, 2001). Genome biologists have studied pathogens with smaller genomes so as to identify the minimum set of genes that are required for virulence, and comparative studies on the genomes of *M. leprae* and *M. tuberculosis* would also help to narrow down the list of genes that are required for virulence of the tubercle bacilli (Vissa *et al.*, 2001). The putative cyclase genes are no exception to the general degeneration of genes observed in the *M. leprae* genome. There are only four genes in the *M. leprae* genome that encode for putative cyclases, viz. ML0201, ML1399, ML1753 and ML2341 (Table 2). There are at least eight identifiable pseudogenes (see

Copyright © 2004 John Wiley & Sons, Ltd. 

Table 2) corresponding to the other cyclases from *M. tuberculosis* (Cole *et al.*, 2001).

### Membrane-bound cyclase

The ML0201 cyclase in *M. leprae* has six transmembrane helices, a HAMP domain and a C-terminal cyclase domain (Figure 6). This is a cyclase similar to those of the Rv1318c cyclase family. However, the enzyme has high sequence similarity to the Rv3645 cyclase (79% identity across the full-length sequences) and is in a region syntenic in *M. tuberculosis*. ML0201 has all the residues required for catalytic activity (Figure 2). Its presence in the genome indicates the importance of the HAMP domain cyclases (alignment shown in Figure 5A) in mycobacteria, since *M. leprae* has retained at least one member from this family.

### Soluble cyclases

Three of the four cyclases in *M. leprae* are predicted to be soluble enzymes (Table 2). The ML1399 cyclase is very similar (74% identical) to the Rv1647 cyclase and lies in a syntenic region of the genome. It possesses all residues required for catalytic activity and substrate selectivity residues are those that classify it to be an adenylyl cyclase (Figure 2).

The ML1753 and ML2341 cyclases are from the interesting family of cyclases that have a NB-ARC domain as a fusion with the cyclase domain (Figures 5C, D and 6). The ML1753 cyclase is the orthologue of *M. tuberculosis* Rv1358, as judged by its position in a syntenic region on the chromosome and domain architecture. However, it lacks the second metal-binding residue and the transition state stabilization residues and therefore might be inactive as a cyclase, much like Rv1358.

The ML2341 cyclase with only a cyclase domain and a NB-ARC domain does not have a corresponding cyclase in *M. tuberculosis* (Figures 5D, 6). The ML2341 cyclase seems to have all the residues required for catalytic activity, except the critical asparagine, and might bind ATP in preference to GTP (Figure 2). This gene lies in a region on the *M. leprae* genome that is flanked by pseudogenes of possibly Rv3728 and Rv3730c in *M. tuberculosis*. However, the Rv3729 gene does not correspond to the ML2341 gene in *M. leprae*.

### Cyclases in related bacteria

### The *Corynebacterium* cyclases

The two *Corynebacterium* genomes analysed here, *C. efficiens* YS-314 and *C. glutamicum* ATCC13032, each have a single putative cyclase

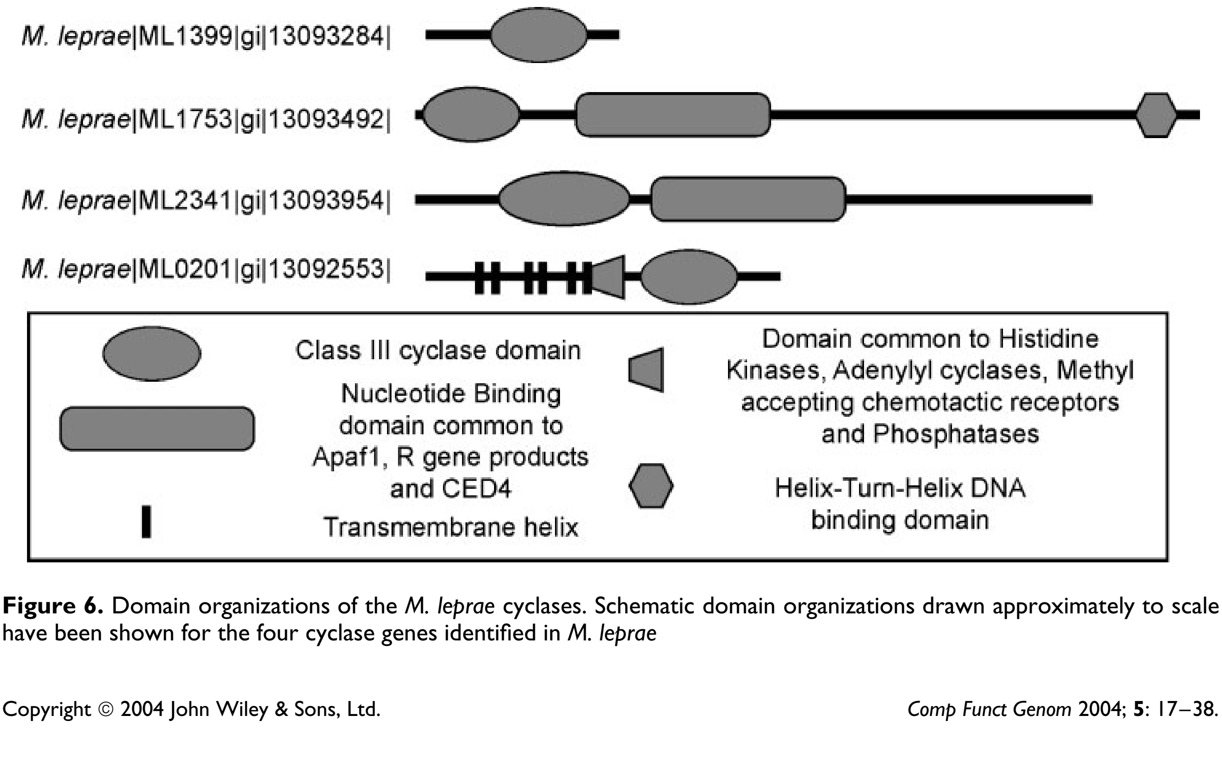

**Figure 6.** Domain organizations of the *M. leprae* cyclases. Schematic domain organizations drawn approximately to scale have been shown for the four cyclase genes identified in *M. leprae*

Copyright © 2004 John Wiley & Sons, Ltd. 

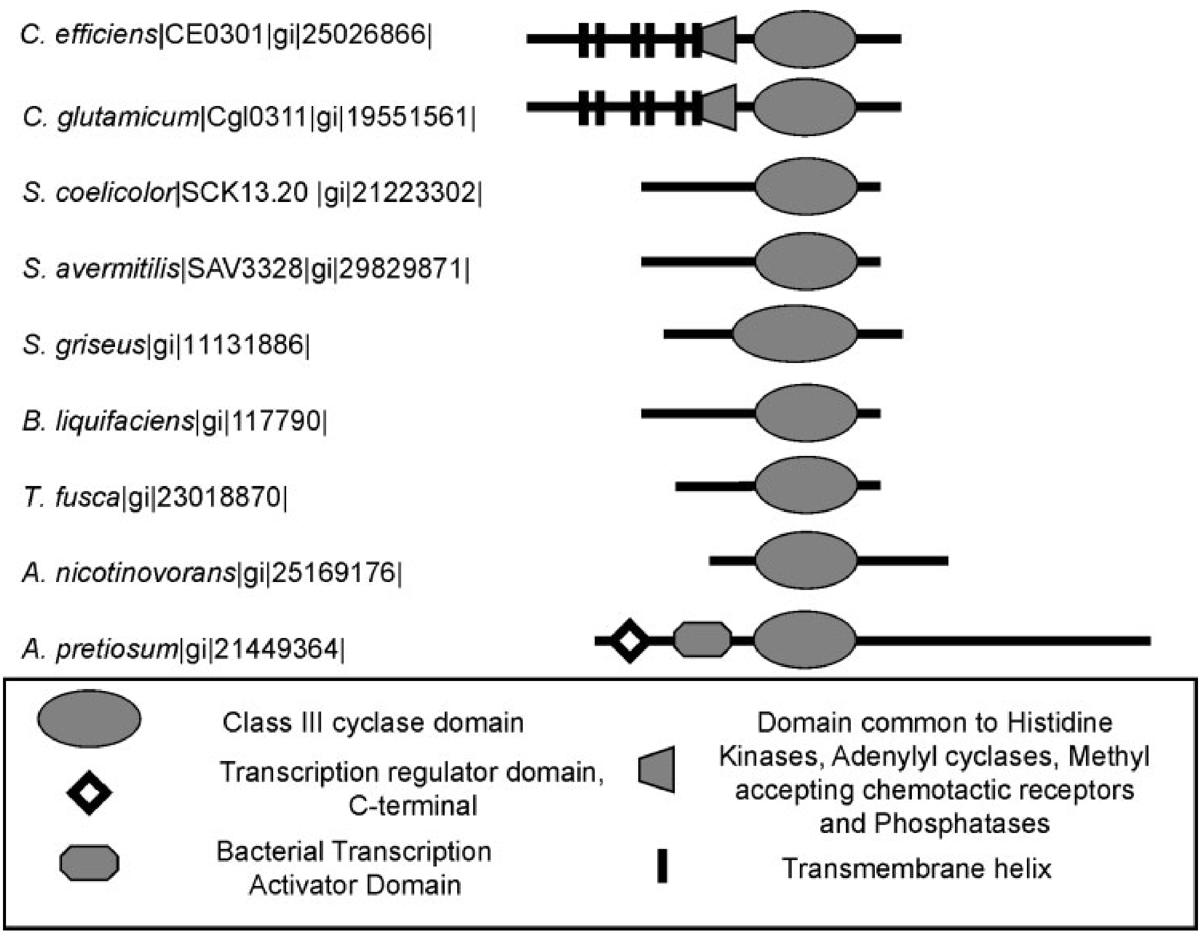

**Figure 7.** Domain organizations of non-mycobacterial cyclases within the Actinobacteria. Schematic domain organizations drawn approximately to scale have been shown for the proteins identified to have a cyclase domain gene (Table 3, Figure 7). These proteins appear to have six transmembrane spanning domains with a HAMP and cyclase domains, and contain all the residues required for cyclase activity (Figures 2, 5A). The proteins are similar (~40% identity) to the Rv3645 cyclase (Figures 3, 5A), and analysis of the region of the genome in these species indicates that these genes are flanked by genes similar to those found adjacent to Rv3645c in *M. tuberculosis,* viz. Rv3646 (DNA topoisomerase) and Rv3644c (subunit of DNA polymerase III). These genes could thus represent orthologues of the Rv3645 cyclase.

### The *Streptomyces* cyclases

The *Streptomyces* are in a separate suborder within the Actinomycetales, the Streptomycineae, unlike the *Corynebacterium* and *Mycobacterium* species, both of which are within the suborder Corynebacterineae (NCBI; **http://www.ncbi.nlm.nih.gov/entrez/query.fcgi?db=Taxonomy**). The genomes of *Streptomyces coelicolor* and *S. avermitilis* reveal the presence of only one cyclase gene each. However, unlike in *Corynebacterium,* these single cyclases are soluble cyclases that cluster with the Rv1264 and Rv2212 cyclases of *M. tuberculosis* (Table 3, Figure 7). A single cyclase each has been found in *S. griseus* and *S. avermitilis*, whose full-length sequences are 66% and 71% identical, respectively, to the cyclase from *S. coelicolor.* However, as seen in the alignment (Figure 2), there is a significant deletion near the second substrate selectivity residue and another insertion in the C-terminus of these cyclases, when compared to all other actinobacterial cyclases. The cyclases from

Copyright © 2004 John Wiley & Sons, Ltd.

*S. coelicolor* (Danchin *et al.*, 1993) and *S. griseus* (Horinouchi *et al.*, 2001) have been shown biochemically to be adenylyl cyclases (Figure 2).

### Other actinobacterial cyclases

We identified cyclase genes through a search of other actinobacterial sequences deposited in nrdb, such as *Brevibacterium liquefaciens, Thermobifida fusca, Arthrobacter nicotinovorans* and *Actinosynnema pretiosum* subsp. *auranticum.* All these cyclases are potential soluble enzymes and, except for the cyclase from *A. pretiosum* subsp. *auranticum,* the other three have only an identifiable cyclase domain (Table 3, Figure 7).

The *T. fusca* cyclase is 39% identical to the cyclase from *B. liquefaciens.* The *Brevibacterium* cyclase has been studied extensively and is activated by pyruvic acid (Lynch *et al.*, 1975; Peters *et al.*, 1991). These cyclases also have a deletion immediately N-terminal to the second substrate selectivity residue but do not have the insertion seen in the *Streptomyces* cyclases. They cluster with the *Streptomyces* cyclases, as seen in Figure 3.

An interesting cyclase was identified in *Arthrobacter nicotinovans,* which is a soil bacterium and in that aspect is like *Streptomyces*. The cyclase domain, although divergent (only 17% identical to the cyclase domain of Rv1625c), has all residues required for catalysis with a conservative substitution (arginine to a lysine residue) of the transition state stabilization residue (Figure 2) and could therefore be active.

The most unique domain organization of any cyclase within the Actinobacteria is probably that of *A. pretiosum* subsp. *auranticum*, which has two additional domains involved in regulation of transcription — a domain similar to the transcription regulatory protein, C-terminal (Pfam Accession No. 00486) and the bacterial transcriptional activator domain (BTAD; Pfam Accession No. 03704; Figure 7). The alignment of this cyclase domain with other cyclases reveals the absence of almost all residues required for catalysis (Figure 2). This protein lies in a region on the genome that is implicated in the biosynthesis of a secondary metabolite from *A. pretiosum* (Yu *et al.*, 2002). This domain is 19% identical to the cyclase domain of Rv1625c. Based on the other domains found in the protein and the known similarity of the class III cyclase domain to the DNA polymerase, this domain might also be a DNA-binding domain.

### Discussion

The presence of different classes of cyclases in a single bacterial genome is rare, and so far only *Pseudomonas aeruginosa* seems to have adenylyl cyclases represented by class I, class II and class III (Yahr *et al.*, 1998; Wolfgang *et al.*, 2003). cAMP was recently shown to regulate virulence pathways in *P. aeruginosa* (Wolfgang *et al.*, 2003). The class III cyclases seem to be far more widely distributed in bacterial genomes (Danchin, 1993; Shenoy *et al.*, 2002). As evident in our study, the Actinobacteria contain only the class III cyclases, and completely lack representatives of all the other five classes of cyclases. Interestingly, only the class III cyclases have been found to exist in multiple copies within bacteria (e.g. *Anabaena* and *Myxococcus)*, as is seen in the eukaryotes (Defer *et al.*, 2000). The number and variations in domain fusions that are seen in the cyclase genes of *Mycobacterium* spp. suggest that this organism could utilize these signalling proteins in its obligate intracellular parasitic lifestyle. No putative cyclase genes are present within any of the regions of difference (Brosch *et al.*, 2001; Cole, 2002) used to classify *M. tuberculosis* strains and related species (Kato-Maeda *et al.*, 2001), suggesting that all organisms in this species retain identical copies of the cyclase genes.

It has been suggested that the class II cyclases originated from the DNA polymerase $\beta$ family and the class III evolved from the DNA polymerase $\alpha$ family (Aravind *et al.*, 1999a). We find here that the cyclase domain exists along with domains seen in subunits of the DNA polymerase, again suggesting a common evolutionary origin for these proteins. The fact that the NB-ARC domain is also found in subunits of the DNA clamp loader (Jaroszewski *et al.*, 2000) has led to the identification of possible new roles for the cyclase domain, such as transcription, as indicated by fusion to domains such as C-terminal transcription regulator, BTAD and HTH DNA-binding domains. The presence of putative inactive cyclase domains, fused along with other domains, also indicates an allosteric regulatory role for the cyclase domain, in being possibly responsive to nucleotide-like molecules.

Copyright © 2004 John Wiley & Sons, Ltd. 

The cyclase from *A. pretiosum* seems to shed some refreshing new light on the role of an inactive cyclase domain in association with domains involved in transcription. The other domains found in this protein are also found in a global regulator of transcription from *Streptomyces,* viz. AfsR (Horinouchi *et al*., 1990), but the *Streptomyces* proteins have a NB-ARC domain positioned in a manner similar to the cyclase domain in the *A. pretiosum* protein (data not shown). This could probably indicate that the cyclase domain substitutes here as an allosteric domain.

All cyclases identified in various actinobacterial genomes have only a single class III cyclase domain in their predicted proteins, unlike several eukaryotic enzymes that contain the C1 and C2 domains fused in tandem. This implies the requirement for homodimerization for the formation of the active site in the bacterial cyclases, if the mechanism of catalysis is similar to that seen in the mammalian enzymes. It would be most interesting to investigate whether there is heterodimerization between similar cyclase domains found in different proteins in bacteria, which could give rise to an even larger repertoire of cyclases with careful regulation of their catalytic activity. Our recent studies on the Rv1625c catalytic domain have also highlighted the subtle changes that can occur at the dimer interface of the two catalytic subunits, and the consequent regulation of its enzyme activity (Shenoy *et al*., 2003).

*M. leprae* seems to have retained a HAMP cyclase and two NB-ARC type cyclases in its genome. The nearest neighbours of the mycobacteria are the corynebacteria, which are within the same suborder. Interestingly, *M. leprae* and the *Corynebacterium* species have retained orthologues of the Rv3645 cyclase of *M. tuberculosis.* The presence of six-transmembrane, HAMP domain-containing cyclases in these genomes suggests that this represents an important type of domain organization of cyclases in these organisms.

Rv1120c seems to be the only cyclase pseudogene in *M. tuberculosis.* We did not identify its corresponding functional or pseudogene in *M. leprae.* It is possible that the gene, if present, has degenerated beyond recognition. Further characterization of this protein and investigation into its transcription and expression would provide information regarding its status as a 'dead' gene or whether it has now gained new functions. Completion of genome sequencing of other *M. tuberculosis* strains and *M. avium* and *M. smegmatis* would also be of value in studying the role of this gene.

The presence of multiple cyclases in complex multigene families has been documented in eukaryotic parasites such as *Trypanosoma* (Alexandre *et al*., 1996; Taylor *et al*., 1999) and *Leishmania* (Sanchez *et al*., 1995). Cyclases are thought to be involved in surface antigen variation in *Trypanosoma* (Pays *et al*., 2001) and also regulate stage-specific developmental phenomena (Alexandre *et al*., 1990; Rolin *et al*., 1993). In fungi, hyphal growth and virulence have been known to also require a class III cyclase, as seen in *Candida albicans* (Rocha *et al*., 2001). It is possible that mycobacteria also use differential expression of cyclases in a stage-specific manner during pathogenesis, as was also suggested after the sequencing of the *M. bovis* genome (Garnier *et al*., 2003). Moreover, the presence of a receptor cyclase (Rv2435c) is interesting in the light of earlier observations of a parasite receptor adenylyl cyclase being activated by peptide molecules and regulating development (Fraidenraich *et al*., 1993).

The cyclase domains found in the Actinobacteria are diverse and form distinct clusters, as seen in Figure 3. There are putative proteins that cluster with typical eukaryotic cyclases, such as Rv1625c and Rv2435c, as well as distant members, such as Rv1647, that appear to be more ancestral. The cyclase domains fused to DNA binding domains appear to be more closely related to the cyclases found in fungi such as *Neurospora* and *Candida,* which again indicates the versatility of the cyclase domain in providing a module that is perhaps able to respond to environmental changes and alter transcription.

Despite the early reports on the presence of cAMP in *Mycobacterium*-infected macrophages, no further information is available on the role of the several enzymes discussed in this study in the organism, including those that have been biochemically characterized from the genome of *M. tuberculosis*. Clearly, research in the direction of elucidation of the biological functions of these enzymes, and cAMP itself, in the pathogen as well as the host would be very fruitful. The adenylyl cyclase activity, which we have measured biochemically in whole cell extracts prepared from *M. tuberculosis* H37Rv, is much higher than that seen in lysates

Copyright © 2004 John Wiley & Sons, Ltd.

of *E. coli* (Shenoy and Visweswariah, unpublished observations). This could reflect the presence of a large number of cyclases in its genome, and possibly that more than one cyclase is expressed at any given time.

In conclusion, the cyclases that are predicted within the Actinobacteria appear to all be adenylyl cyclases, and we have not identified any guanylyl cyclases, based on the substrate selectivity residues identified in mammalian class III cyclases. The cyclase genes that we have identified here thus appear to encode a large and an interesting group of proteins that might be involved in important signalling events. Further biochemical and structural studies of these proteins present in Actinobacteria might improve our understanding of the biology of several important pathogenic bacteria.

### Acknowledgements

This work was supported by financial assistance from the Wellcome Trust, UK, and the Department of Biotechnology, Government of India. NS is supported by the International Senior Fellowship Programme on Biomedical Sciences by the Wellcome Trust, UK.

### References

Alexandre S, Paindavoine P, Hanocq-Quertier J, *et al.* 1996. Families of adenylate cyclase genes in *Trypanosoma brucei*. *Mol Biochem Parasitol* **77**: 173–182.

Alexandre S, Paindavoine P, Tebabi P, *et al.* 1990. Differential expression of a family of putative adenylate/guanylate cyclase genes in *Trypanosoma brucei*. *Mol Biochem Parasitol* **43**: 279–288.

Altschul SF, Madden TL, Schaffer AA, *et al.* 1997. Gapped BLAST and PSI-BLAST: a new generation of protein database search programs. *Nucleic Acids Res* **25**: 3389–3402.

Appleman JA, Stewart V. 2003. Mutational analysis of a conserved signal-transducing element: the HAMP linker of the *Escherichia coli* nitrate sensor NarX. *J Bacteriol* **185**: 89–97.

Aravind L, Dixit VM, Koonin EV. 2001. Apoptotic molecular machinery: vastly increased complexity in vertebrates revealed by genome comparisons. *Science* **291**: 1279–1284.

Aravind L, Koonin EV. 1999a. DNA polymerase $\beta$-like nucleotidyltransferase superfamily: identification of three new families, classification and evolutionary history. *Nucleic Acids Res* **27**: 1609–1618.

Aravind L, Ponting CP. 1999b. The cytoplasmic helical linker domain of receptor histidine kinase and methyl-accepting proteins is common to many prokaryotic signalling proteins. *FEMS Microbiol Lett* **176**: 111–116.

Bateman A, Birney E, Cerruti L, *et al.* 2002. The Pfam protein families database. *Nucleic Acids Res* **30**: 276–280.

Bentley SD, Chater KF, Cerdeno-Tarraga AM, *et al.* 2002. Complete genome sequence of the model actinomycete *Streptomyces coelicolor* A3(2). *Nature* **417**: 141–147.

Bentley SD, Maiwald M, Murphy LD, *et al.* 2003. Sequencing and analysis of the genome of the Whipple's disease bacterium *Tropheryma whipplei*. *Lancet* **361**: 637–644.

Bieger B, Essen LO. 2001. Structural analysis of adenylate cyclases from *Trypanosoma brucei* in their monomeric state. *EMBO J* **20**: 433–445.

Brosch R, Pym AS, Gordon SV, *et al.* 2001. The evolution of mycobacterial pathogenicity: clues from comparative genomics. *Trends Microbiol* **9**: 452–458.

Cases I, de Lorenzo V. 1998. Expression systems and physiological control of promoter activity in bacteria. *Curr Opin Microbiol* **1**: 303–310.

CDART. Conserved Domain Architecture Retrieval Tool. **http://www.ncbi.nlm.nih.gov/Structure/lexington/lexington.cgi?cmd = rps**.

Cole ST. 2002. Comparative and functional genomics of the *Mycobacterium tuberculosis* complex. *Microbiology* **148**: 2919–2928.

Cole ST, Brosch R, Parkhill J, *et al.* 1998. Deciphering the biology of *Mycobacterium tuberculosis* from the complete genome sequence. *Nature* **393**: 537–544.

Cole ST, Eiglmeier K, Parkhill J, *et al.* 2001. Massive gene decay in the leprosy bacillus. *Nature* **409**: 1007–1011.

Confer DL, Eaton JW. 1982. Phagocyte impotence caused by an invasive bacterial adenylate cyclase. *Science* **217**: 948–950.

Cotta MA, Whitehead TR, Wheeler MB. 1998. Identification of a novel adenylate cyclase in the ruminal anaerobe, *Prevotella ruminicola* D31d. *FEMS Microbiol Lett* **164**: 257–260.

Danchin A. 1993. Phylogeny of adenylyl cyclases. *Adv Second Messenger Phosphoprotein Res* **27**: 109–162.

Danchin A, Pidoux J, Krin E, *et al.* 1993. The adenylate cyclase catalytic domain of *Streptomyces coelicolor* is carboxy-terminal. *FEMS Microbiol Lett* **114**: 145–151.

Defer N, Best-Belpomme M, Hanoune J. 2000. Tissue specificity and physiological relevance of various isoforms of adenylyl cyclase. *Am J Physiol Renal Physiol* **279**: F400–416.

Drum CL, Yan SZ, Bard J, *et al.* 2002. Structural basis for the activation of anthrax adenylyl cyclase exotoxin by calmodulin. *Nature* **415**: 396–402.

Eddy SR. 2001. HMMER: Profile hidden Markov models for biological sequence analysis. **http://hmmer.wustl.edu/**.

Fleischmann RD, Alland D, Eisen JA, *et al.* 2002. Whole-genome comparison of *Mycobacterium tuberculosis* clinical and laboratory strains. *J Bacteriol* **184**: 5479–5490.

Fraidenraich D, Pena C, Isola EL, *et al.* 1993. Stimulation of *Trypanosoma cruzi* adenylyl cyclase by an $\alpha$D-globin fragment from *Triatoma* hindgut: effect on differentiation of epimastigote to trypomastigote forms. *Proc Natl Acad Sci USA* **90**: 10 140–10 144.

Galperin MY, Nikolskaya AN, Koonin EV. 2001. Novel domains of the prokaryotic two-component signal transduction systems. *FEMS Microbiol Lett* **203**: 11–21.

Garnier T, Eiglmeier K, Camus JC, *et al.* 2003. The complete genome sequence of *Mycobacterium bovis*. *Proc Natl Acad Sci USA*.

Gobel M, Kassel-Cati K, Schmidt E, *et al.* 2002. Degradation of aromatics and chloroaromatics by *Pseudomonas sp.* strain B13: cloning, characterization, and analysis of sequences encoding

Copyright © 2004 John Wiley & Sons, Ltd. 

3-oxoadipate : succinyl-coenzyme A (CoA) transferase and 3-oxoadipyl-CoA thiolase. *J Bacteriol* **184**: 216–223.

Guo YL, Seebacher T, Kurz U, *et al.* 2001. Adenylyl cyclase Rv1625c of *Mycobacterium tuberculosis*: a progenitor of mammalian adenylyl cyclases. *EMBO J* **20**: 3667–3675.

Hannenhalli SS, Russell RB. 2000. Analysis and prediction of functional sub-types from protein sequence alignments. *J Mol Biol* **303**: 61–76.

Hoover DL, Friedlander AM, Rogers LC, *et al.* 1994. Anthrax edema toxin differentially regulates lipopolysaccharide-induced monocyte production of tumor necrosis factor $\alpha$ and interleukin-6 by increasing intracellular cyclic AMP. *Infect Immun* **62**: 4432–4439.

Horinouchi S, Kito M, Nishiyama M, *et al.* 1990. Primary structure of AfsR, a global regulatory protein for secondary metabolite formation in *Streptomyces coelicolor* A3(2). *Gene* **95**: 49–56.

Horinouchi S, Ohnishi Y, Kang DK. 2001. The A-factor regulatory cascade and cAMP in the regulation of physiological and morphological development in *Streptomyces griseus*. *J Ind Microbiol Biotechnol* **27**: 177–182.

Jaroszewski L, Rychlewski L, Reed JC, *et al.* 2000. ATP-activated oligomerization as a mechanism for apoptosis regulation: fold and mechanism prediction for CED-4. *Proteins* **39**: 197–203.

Jeanmougin F, Thompson JD, Gouy M, *et al.* 1998. Multiple sequence alignment with Clustal X. *Trends Biochem Sci* **23**: 403–405.

Kato-Maeda M, Rhee JT, Gingeras TR, *et al.* 2001. Comparing genomes within the species *Mycobacterium tuberculosis*. *Genome Res* **11**: 547–554.

Kumar S, Tamura K, Jakobsen IB, *et al.*, 2001. *MEGA2: Molecular Evolutionary Genetics Analysis Software*. Arizona State University: Arizona, USA.

Leppla SH. 1982. Anthrax toxin edema factor: a bacterial adenylate cyclase that increases cyclic AMP concentrations of eukaryotic cells. *Proc Natl Acad Sci USA* **79**: 3162–3166.

Letunic I, Goodstadt L, Dickens NJ, *et al.* 2002. Recent improvements to the SMART domain-based sequence annotation resource. *Nucleic Acids Res* **30**: 242–244.

Linder JU, Schultz A, Schultz JE. 2002. Adenylyl cyclase Rv1264 from *Mycobacterium tuberculosis* has an autoinhibitory N-terminal domain. *J Biol Chem* **277**: 15 271–15 276.

Liu Y, Ruoho AE, Rao VD, *et al.* 1997. Catalytic mechanism of the adenylyl and guanylyl cyclases: modeling and mutational analysis. *Proc Natl Acad Sci USA* **94**: 13 414–13 419.

Lowrie DB, Aber VR, Jackett PS. 1979. Phagosome-lysosome fusion and cyclic adenosine 3′ : 5′-monophosphate in macrophages infected with *Mycobacterium microti, Mycobacterium bovis* BCG or *Mycobacterium lepraemurium*. *J Gen Microbiol* **110**: 431–441.

Lowrie DB, Jackett PS, Ratcliffe NA. 1975. *Mycobacterium microti* may protect itself from intracellular destruction by releasing cyclic AMP into phagosomes. *Nature* **254**: 600–602.

Lynch TJ, Tallant EA, Cheung WY. 1975. *Brevibacterium liquefaciens* adenylate cyclase and its *in vivo* stimulation by pyruvate. *J Bacteriol* **124**: 1106–1112.

McCue LA, McDonough KA, Lawrence CE. 2000. Functional classification of cNMP-binding proteins and nucleotide cyclases with implications for novel regulatory pathways in *Mycobacterium tuberculosis*. *Genome Res* **10**: 204–219.

Mowbray SL, Sandgren MO. 1998. Chemotaxis receptors: a progress report on structure and function. *J Struct Biol* **124**: 257–275.

NCBI. FTP site for genomes. **ftp://ftp.ncbi.nih.gov/genomes/Bacteria/**.

NCBI. NCBI BLAST Home Page. **http://www.ncbi.nlm.nih.gov/BLAST/**.

NCBI. RPS-BLAST CD-search databases. **ftp://ftp.ncbi.nih.gov/pub/mmdb/cdd/**.

NCBI. Taxonomy. **http://www.ncbi.nlm.nih.gov/entrez/query.fcgi?db = Taxonomy**.

Neuwald AF, Aravind L, Spouge JL, *et al.* 1999. AAA+: A class of chaperone-like ATPases associated with the assembly, operation, and disassembly of protein complexes. *Genome Res* **9**: 27–43.

Notredame C, Higgins DG, Heringa J. 2000. T-Coffee: a novel method for fast and accurate multiple sequence alignment. *J Mol Biol* **302**: 205–217.

Ollis DL, Cheah E, Cygler M, *et al.* 1992. The $\alpha/\beta$ hydrolase fold. *Protein Eng* **5**: 197–211.

Omura S, Ikeda H, Ishikawa J, *et al.* 2001. Genome sequence of an industrial microorganism *Streptomyces avermitilis*: deducing the ability of producing secondary metabolites. *Proc Natl Acad Sci USA* **98**: 12 215–12 220.

Pays E, Lips S, Nolan D, *et al.* 2001. The VSG expression sites of *Trypanosoma brucei:* multipurpose tools for the adaptation of the parasite to mammalian hosts. *Mol Biochem Parasitol* **114**: 1–16.

Peters EP, Wilderspin AF, Wood SP, *et al.* 1991. A pyruvate-stimulated adenylate cyclase has a sequence related to the fes/fps oncogenes and to eukaryotic cyclases. *Mol Microbiol* **5**: 1175–1181.

Pfam. Protein Family Database. **http://www.sanger.ac.uk/Software/Pfam/**.

Reddy SK, Kamireddi M, Dhanireddy K, *et al.* 2001. Eukaryotic-like adenylyl cyclases in *Mycobacterium tuberculosis* H37Rv: cloning and characterization. *J Biol Chem* **276**: 35 141–35 149.

Rocha CR, Schroppel K, Harcus D, *et al.* 2001. Signaling through adenylyl cyclase is essential for hyphal growth and virulence in the pathogenic fungus *Candida albicans*. *Mol Biol Cell* **12**: 3631–3643.

Rolin S, Paindavoine P, Hanocq-Quertier J, *et al.* 1993. Transient adenylate cyclase activation accompanies differentiation of *Trypanosoma brucei* from bloodstream to procyclic forms. *Mol Biochem Parasitol* **61**: 115–125.

Sanchez MA, Zeoli D, Klamo EM, *et al.* 1995. A family of putative receptor-adenylate cyclases from *Leishmania donovani*. *J Biol Chem* **270**: 17 551–17 558.

Schaffer AA, Wolf YI, Ponting CP, *et al.* 1999. IMPALA: matching a protein sequence against a collection of PSI-BLAST-constructed position-specific score matrices. *Bioinformatics* **15**: 1000–1011.

Schell MA, Karmirantzou M, Snel B, *et al.* 2002. The genome sequence of *Bifidobacterium longum* reflects its adaptation to the human gastrointestinal tract. *Proc Natl Acad Sci USA* **99**: 14 422–14 427.

Shenoy AR, Srinivasan N, Subramaniam M, *et al.* 2003. Mutational analysis of the *Mycobacterium tuberculosis* Rv1625c adenylyl cyclase: residues that confer nucleotide specificity contribute to dimerization. *FEBS Lett* **545**: 253–259.

Copyright © 2004 John Wiley & Sons, Ltd. 

Shenoy AR, Srinivasan N, Visweswariah SS. 2002. The ascent of nucleotide cyclases: conservation and evolution of a theme. *J Biosci* **27**: 85–91.

Sherman DR, Voskuil M, Schnappinger D, *et al.* 2001. Regulation of the *Mycobacterium tuberculosis* hypoxic response gene encoding $\alpha$-crystallin. *Proc Natl Acad Sci USA* **98**: 7534–7539.

Sismeiro O, Trotot P, Biville F, *et al.* 1998. *Aeromonas hydrophila* adenylyl cyclase 2: a new class of adenylyl cyclases with thermophilic properties and sequence similarities to proteins from hyperthermophilic archaebacteria. *J Bacteriol* **180**: 3339–3344.

SMART. Simple Modular Architechture Research Tool. **http://smart.embl-heidelberg.de/**.

Sunahara RK, Beuve A, Tesmer JJ, *et al.* 1998. Exchange of substrate and inhibitor specificities between adenylyl and guanylyl cyclases. *J Biol Chem* **273**: 16 332–16 338.

Susstrunk U, Pidoux J, Taubert S, *et al.* 1998. Pleiotropic effects of cAMP on germination, antibiotic biosynthesis and morphological development in *Streptomyces coelicolor*. *Mol Microbiol* **30**: 33–46.

Tang WJ, Hurley JH. 1998. Catalytic mechanism and regulation of mammalian adenylyl cyclases. *Mol Pharmacol* **54**: 231–240.

Tatusov RL, Natale DA, Garkavtsev IV, *et al.* 2001. The COG database: new developments in phylogenetic classification of proteins from complete genomes. *Nucleic Acids Res* **29**: 22–28.

Taylor MC, Muhia DK, Baker DA, *et al.* 1999. *Trypanosoma cruzi* adenylyl cyclase is encoded by a complex multigene family. *Mol Biochem Parasitol* **104**: 205–217.

Tellez-Sosa J, Soberon N, Vega-Segura A, *et al.* 2002. The *Rhizobium etli* cyaC product: characterization of a novel adenylate cyclase class. *J Bacteriol* **184**: 3560–3568.

Tesmer JJ, Sprang SR. 1998. The structure, catalytic mechanism and regulation of adenylyl cyclase. *Curr Opin Struct Biol* **8**: 713–719.

Tesmer JJ, Sunahara RK, Gilman AG, *et al.* 1997. Crystal structure of the catalytic domains of adenylyl cyclase in a complex with Gs$\alpha$.GTP$\gamma$S. *Science* **278**: 1907–1916.

TIGR. Operon Information. **http://www.tigr.org/tigr-scripts/operons/pairs.cgi?taxon_id=89**.

TIGR. Unfinished Genomes. **http://www.tigr.org/tigr-scripts/ufmg/ReleaseDate.pl**.

TMHMM. TMHMM Server, v.2.0. **http://www.cbs.dtu.dk/services/TMHMM/**.

Tucker CL, Hurley JH, Miller TR, *et al.* 1998. Two amino acid substitutions convert a guanylyl cyclase, RetGC-1, into an adenylyl cyclase. *Proc Natl Acad Sci USA* **95**: 5993–5997.

van der Biezen EA, Jones JD. 1998. The NB-ARC domain: a novel signalling motif shared by plant resistance gene products and regulators of cell death in animals. *Curr Biol* **8**: R226–227.

Vissa VD, Brennan PJ. 2001. The genome of *Mycobacterium leprae:* a minimal mycobacterial gene set. *Genome Biol* **2**: REVIEWS1023.

Wedel B, Garbers D. 2001. The guanylyl cyclase family at Y2K. *Annu Rev Physiol* **63**: 215–233.

Weiss AA, Hewlett EL, Myers GA, *et al.* 1984. Pertussis toxin and extracytoplasmic adenylate cyclase as virulence factors of *Bordetella pertussis*. *J Infect Dis* **150**: 219–222.

Wirth JJ, Kierszenbaum F. 1982. Inhibitory action of elevated levels of adenosine-3′ : 5′-cyclic monophosphate on phagocytosis: effects on macrophage–*Trypanosoma cruzi* interaction. *J Immunol* **129**: 2759–2762.

Wolfgang MC, Lee VT, Gilmore ME, *et al.* 2003. Coordinate regulation of bacterial virulence genes by a novel adenylate cyclase-dependent signaling pathway. *Dev Cell* **4**: 253–263.

Yahr TL, Vallis AJ, Hancock MK, *et al.* 1998. ExoY, an adenylate cyclase secreted by the *Pseudomonas aeruginosa* type III system. *Proc Natl Acad Sci USA* **95**: 13 899–13 904.

Yan SZ, Huang ZH, Shaw RS, *et al.* 1997. The conserved asparagine and arginine are essential for catalysis of mammalian adenylyl cyclase. *J Biol Chem* **272**: 12 342–12 349.

Yeats C, Bentley S, Bateman A. 2003. New knowledge from old: *in silico* discovery of novel protein domains in *Streptomyces coelicolor*. *BMC Microbiol* **3**: 3.

Yu TW, Bai L, Clade D, *et al.* 2002. The biosynthetic gene cluster of the maytansinoid antitumor agent ansamitocin from *Actinosynnema pretiosum*. *Proc Natl Acad Sci USA* **99**: 7968–7973.

Zimmermann G, Zhou D, Taussig R. 1998. Mutations uncover a role for two magnesium ions in the catalytic mechanism of adenylyl cyclase. *J Biol Chem* **273**: 19 650–19 655.

Copyright © 2004 John Wiley & Sons, Ltd. 
